# Oxygen-Mediated Structural Modulation and Ion Transport in *x*Na_2_O‑TaCl_5_ Glass Electrolytes

**DOI:** 10.1021/jacs.5c10564

**Published:** 2025-11-11

**Authors:** Zheng Huang, Neha Yadav, Shun Itakura, Peng Song, Hirofumi Akamatsu, Katsuro Hayashi, Prashun Gorai, Saneyuki Ohno

**Affiliations:** a Institute of Multidisciplinary Research for Advanced Materials, 13101 Tohoku University 2-1-1 Katahira, Aoba-ku, Sendai, Miyagi 980-8577, Japan; b Department of Frontier Sciences for Advanced Environment, Graduate School of Environmental Studies, 13101 Tohoku University, 2-1-1 Katahira, Aoba-ku, Sendai, Miyagi 980-8577, Japan; c Department of Applied Chemistry, Graduate School of Engineering, 12923 Kyushu University, 744 Motooka, Nishi-ku, Fukuoka 819-0395, Japan; d Department of Metallurgical and Materials Engineering, 3557 Colorado School of Mines, Golden, Colorado 80401, United States; e Chemical & Biological Engineering, 8024 Rensselaer Polytechnic Institute, Troy, New York 12180, United States

## Abstract

Understanding the structure–property relationship in glass solid electrolytes (SEs) remains a major challenge due to their inherent disorder and the difficulty of probing local structures, particularly in relation to oxygen incorporation. Despite recent interest in multianion halide solid electrolytes, there are few systematic studies on how varying the oxygen content affects the local structure and ion transport. Here, we investigate a series of amorphous sodium oxychloride SEs with the composition *x*Na_2_O-TaCl_5_ (0.1 ≤ *x* ≤ 1.5), revealing three distinct conductivity regimes and achieving a maximum of 4.1 mS cm^–1^ at room temperature. Synchrotron and lab X-ray total scattering and Raman spectroscopy indicate the gradual formation of Ta–O–Ta bonds that bridge the two or more metal chloride polyhedra, while *ab initio* molecular dynamics simulations clarify the distinct roles of bridging and nonbridging O^2–^ species. These findings not only provide mechanistic insights into oxygen-mediated glass formation but also establish guiding principles for multianion engineering in the design of next-generation solid electrolytes.

## Graphical abstract

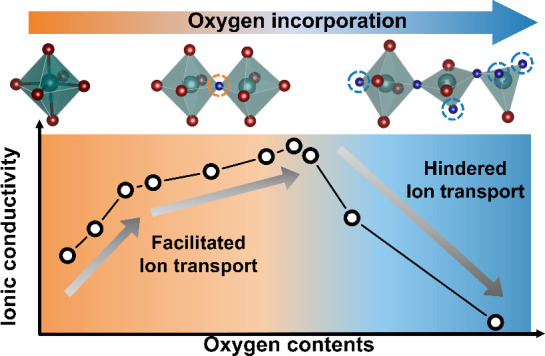

## Introduction

Solid-state sodium-ion (Na-ion) batteries are promising candidates for next-generation energy storage technology owing to their improved safety and the elemental abundance of sodium.^1,2^ To realize solid-state Na-ion batteries, highly conductive, (electro)­chemically stable, highly reproducible, and easily processable Na-ion conducting solid electrolytes (SEs) are needed.^3–7^ To date, however, none of the known SEs fully meet all the requirements, which has motivated further materials development. Over the past few years, halide SEs, especially chloride SEs, have emerged and gained widespread attention. With higher polarizability of Cl^–^ over that of O^2–^ and its superior oxidation stability compared to S^2–^, besides the high deformability of halides,^8,9^ numerous experimental and theoretical efforts have been devoted to lithium-ion (Li-ion) conducting halides to develop highly conductive SEs.^8,10–15^ Inspired by the success of Li-ion conducting halides, Na-ion conducting analogues, e.g., Na_3–*x*_M_1–*x*_Zr_*x*_Cl_6_ (M = Y, Er, and In),^16–18^ Na_1+*x*_M_1–*x*_Zr_*x*_Cl_6_ (M = Nb and Ta),^19^ and (1–*x*)­[Na_0.75_M_1.75_Cl_6_]·*x*[NaTaCl_6_] (M = La and Sm),^20^ have also been explored; however, despite chemical similarity, their ionic conductivities remain significantly lower than those of their Li-ion counterparts. While the influence of carrier concentration,^16–18^ inductive effect,^19^ crystallinity,^21^ and structural heterogeneity^20^ on ion transport properties has been discussed through cation substitution strategies, new strategies to drastically improve ionic conductivity are needed.

A fundamentally new strategy, utilizing multiple anions in the anionic framework, has recently demonstrated multifold improvements in the ionic conductivity of halides. Originating from the report of LiTaOCl_4_ and LiNbOCl_4_ in 2023, achieving room-temperature ionic conductivity >10 mS cm^–1^,^22^ Li-ion conducting oxyhalides, e.g., AAlCl_2.5_O_0.75_ (A = Li and Na),^23^ Li_2+2*x*_ZrCl_4_O_1+__x_,^24^ and *x*Li_2_O-MCl_5_ (M = Ta and Hf),^25^ are in the spotlight for their outstanding ionic conductivity at room temperature.^23–25^ Naturally, the same strategy has been employed in Na-ion conducting halides and successfully demonstrated a boost in ionic conductivity. For instance, the melt-quenched glass SEs of 0.5Na_2_O_2_–TaCl_5_ exhibit ionic conductivity higher than NaTaCl_6_ by a factor of 88.^19,26^ Subsequently, NaTaOCl_4_, NaNbCl_6–2*x*_O_*x*_, Na_1+5*x*_TaCl_6–5*x*_O_5*x*_, and Na_2_O_2_-MCl_5_ (M = Hf, Zr, and Ta) have also been revealed to possess fast Na-ion transport.^27–31^

Nevertheless, the mechanistic origin underlying the strong enhancement of ionic conductivity in glass oxychlorides upon incorporation of O^2–^, despite its high electronegativity and larger valence, which are typically associated with increased migration barriers and reduced ion mobility, remains elusive. In the crystalline phase, such as LiNbOCl_4_, which consists of one-dimensional chains of edge-sharing [NbO_2_Cl_4_]^−^ octahedra bridged by O^2–^, molecular dynamics simulations have suggested that the flexibility of the NbCl_4_ plane (perpendicular to the chain direction) can flatten the energy landscape for mobile cations.^32^ In contrast, in the glass phases, the enhanced ionic conductivity has been attributed to the amorphous nature of the multianion network as exemplified in *x*Li_2_O-MCl_*y*_ (M = Ta or Hf, 0.8 ≤ *x* ≤ 2, *y* = 5 or 4) and Na_2_O_2_–MCl_*y*_ (M = Hf, Zr, and Ta; *y* = 4 or 5).^25,29^ The introduction of O^2–^ facilitates the structural rearrangements that promote glass formation, leading to a significantly broader distribution of the local polyhedral environments, e.g., [MCl_4_O]^−^ and [MCl_5–*a*_O_*a*_]^*a*−^ (2 ≤ *a* ≤ 5). Within this framework, O^2–^ is proposed to play a dual role: bridging adjacent octahedra to stabilize the amorphous structure and occupying the nonbridging positions of the octahedra to generate a relatively open framework, thereby enhancing Na-ion transport. However, the presence of O^2–^ exposed to the transport pathway could also increase Coulomb interaction with migrating cations, which would typically hinder ion transport and seemingly contradict the observed conductivity enhancement. Furthermore, while the number of studies reporting transport properties in these materials is increasing, detailed insights into the corresponding local structural evolution as a function of the O^2–^ contents remain limited. This gap is likely due to the experimental challenges and restricted beamtime availability for advanced structural characterization techniques applicable to disordered materials.

Motivated by these considerations, a series of Na-ion conducting oxychlorides with the composition *x*Na_2_O-TaCl_5_ (0.1 ≤ *x* ≤ 1.5) is synthesized with systematic variation of the oxygen content. Amorphous phases were obtained over a broad compositional range, accompanied by distinct changes in ionic conductivity, which can be categorized into three compositional regimes: (1) substantial enhancement for *x* ≤ 0.2; (2) a gradual increase with an increasing Na content in the range 0.2 ≤ *x* ≤ 0.8; and (3) a sharp drop in conductivity beyond *x* > 0.8, associated with an increase in the migration barrier. The local structural evolution is investigated for all synthesized samples using synchrotron and laboratory X-ray total scattering along with Raman spectroscopy. These analyses reveal the emergence and monotonic growth of a peak corresponding to the Ta–Ta distance in tetrahedral units bridged by O^2–^ in Ta–O–Ta coordination. To gain further insights into the local structure of obtained glasses, *ab initio* molecular dynamics (AIMD) simulations are performed with various O^2–^ contents, enabling quantification of the ratio of bridging to nonbridging O^2–^ species. In relation to the observed transport behavior, we highlight the crucial role of bridging O^2–^, which does not exhibit significant interaction with migrating Na ions, in contrast to nonbridging O^2–^, which exerts stronger interactions and impedes ion mobility. As a result, by optimizing the ratio of bridging to nonbridging O^2–^ and the Na-ion coordination environment and free volume through oxygen content control, a high ionic conductivity of 4.1 mS cm^–1^ is achieved.

## Results and Discussion

### Glass Formation with Various Oxygen Contents and Its Impact on Ionic Conductivity

The series of Na-ion conducting oxyhalides was mechanochemically synthesized from starting materials Na_2_O and TaCl_5_. Since the presence of ca. 10 mol % Na_2_O_2_ in the Na_2_O precursor was confirmed by Rietveld refinement, thereby, the actual sodium and oxygen contents of the synthesized samples slightly deviate from the nominal composition (Figure 1a). For a more precise measure of the oxygen content, the actual nominal composition was calculated as *x*_A_Na_2_O_1.1_-TaCl_5_ (*x*_A_ = 0.1, 0.21, 0.31, 0.52, 0.72, 0.82, 0.88, 1.03, and 1.55). The details of the actual compositions are described in the Supporting Information (Table S1). Nonetheless, as this deviation does not affect the discussion on the influence of oxygen content on the structure and ion transport properties, we use the actual composition *x*_A_ in the presented data unless specified hereafter. The elemental analysis, as shown in Table S2, using energy-dispersive X-ray spectroscopy (EDS), confirms the atomic fractions of Na, Ta, and Cl and no significant contamination from the milling media (ZrO_2_). Oxygen incorporation was also corroborated, but a reliable quantification of oxygen contents through EDS is generally challenging.^27^

**Figure 1:**
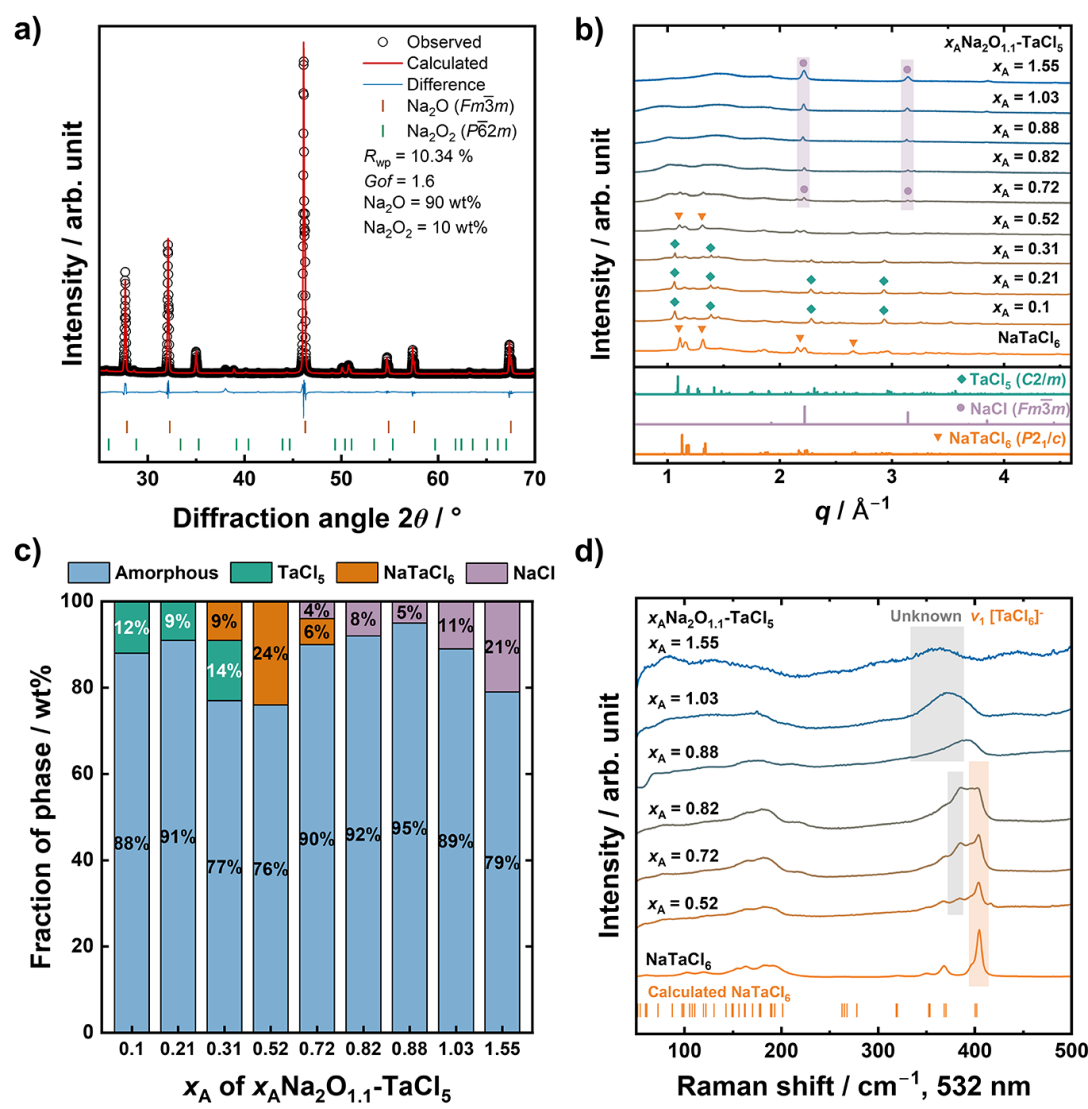
(a) Rietveld fit of the Na_2_O precursor. Due to the presence of a 10 wt % Na_2_O_2_ impurity, the compositions of the synthesized samples were corrected and are referred to as *x*_A_Na_2_O_1.1_-TaCl_5_. (b) Comparison of lab X-ray diffraction patterns of *x*_A_Na_2_O_1.1_-TaCl_5_ samples. (c) Phase fractions of amorphous and crystalline components estimated using the relative intensity ratio method with 10 wt % of Si powder as an internal standard. (d) Raman spectra of *x*_A_Na_2_O_1.1_-TaCl_5_. The sharp peaks at ∼415 cm^–1^ correspond to the stretching vibration of TaCl_6_^–^ units.^19^ This peak is retained up to *x*_A_ = 0.82, as highlighted by the orange shading. With an increase in Na_2_O, additional vibrational modes emerge near ∼380 cm^–1^, indicating the evolution of local structural motifs.

The powder X-ray diffraction (XRD) patterns of the synthesized samples are shown in Figure 1b. Compared with the XRD pattern of Na-ion conducting halide NaTaCl_6_, the broad halo pattern indicating the formation of an amorphous phase can be observed in all oxyhalide samples *x*_A_Na_2_O_1.1_-TaCl_5_. Even though the crystallinity of NaTaCl_6_ is known to decrease under extreme ball milling conditions,^19,21^ incorporation of oxygen as an additional anion, even only minute amounts, still significantly promotes the formation of the amorphous phase. Besides the broad amorphous halo, small diffraction peaks from crystalline phases can also be observed in the XRD patterns, which vary with the composition. When the oxygen contents are low (*x*_A_ ≤ 0.31), diffraction peaks from crystalline impurities could be indexed to the raw material TaCl_5_. As *x*_A_ increased to 0.52, the peaks of TaCl_5_ disappeared, and the monoclinic crystal of NaTaCl_6_ with the space group *P*2_1_/*c* can be observed in XRD patterns. Further increasing *x*_A_ led to the disappearance of NaTaCl_6_ peaks and the appearance of NaCl peaks. The phase fractions of the amorphous phase and crystalline side phases were quantified by the relative intensity ratio (RIR) analysis and are shown in Figure 1c, confirming that the amorphous phase is the dominant phase. The technical details of RIR analysis are provided in the Supporting Information. Notably, after verification of the Na_2_O contents in such a wide range, there are still no crystalline diffraction peaks containing oxygen that were detected even in the extremely Na_2_O-rich sample with *x*_A_ = 1.55. This suggests that the oxygen content in the amorphous phase is successfully tuned, which enables further investigation into the effects of oxygen substitution on the local structure and ion transport properties.

Since diffraction-based structural analysis is hindered by the highly amorphous nature of oxyhalide samples, Raman spectroscopy was used to capture the local structural changes with varying oxygen content. To better understand the influence of oxygen, the vibrational signal of the monoanionic halide NaTaCl_6_ was also measured as a reference. As a result, the Raman spectra of NaTaCl_6_ match well with the calculated vibrational model (Figure 1d), consistent with our previous work.^19^ The intense peak at ∼415 cm^–1^ (marked in orange) originates from the stretching vibrational modes of isolated TaCl_6_^–^ octahedra. This signal broadens but remains observable up to *x*_A_ = 0.82 and then vanishes in samples with *x*_A_ = 0.88, 1.03, and 1.55. In addition, new vibrational signals at ∼380 cm^–1^ appear in *x*_A_Na_2_O_1.1_-TaCl_5_ samples (marked in gray). The relative intensity of the new signals increases with *x*_A_ and shifts to the low Raman shift side above *x*_A_ = 0.88. Considering that impurity-phase NaCl has no characteristic vibrational signal during 300–500 cm^–1^ (Figure S3c), such a change in Raman spectra should originate from the vibrational model change in the amorphous phase, consistent with the above discussed compositional change. In the Li_2_O/Li_2_O_2_-AlCl_3_ series, the incorporation of oxygen has also been reported to give rise to new Raman signals, which were assigned to the vibrational mode of formed Al–O bonds.^33^ By analogy, the new signal at ∼380 cm^–1^ observed in our samples can likely be attributable to the vibrations of multianion polyhedra formed upon oxygen incorporation. When *x*_A_ = 1.55, the whole Raman spectrum flattens. This change in the Raman spectra was observed in *x*Li_2_O-TaCl_5_ with a high oxygen content,^25^ which was attributed to the complex vibrational model in a highly disordered local structure. Notably, no discernible peaks were detected in the 600–900 cm^–1^ region in this study indicating the absence of remaining peroxide ions in the matrix (Figure S3a,b), whereas the previous studies found peaks and attributed them to Ta–O vibrational modes by referencing Ta_2_O_5_.^25,26,34^ Combining the results of XRD and Raman spectroscopy, the incorporation of oxygen decreases the fraction of isolated TaCl_6_^–^ octahedra and promotes the formation of the amorphous phase, whose local structure changes with oxygen contents.

To investigate the relationship between the oxygen content and ion transport properties, temperature-dependent electrochemical impedance spectroscopy (EIS) was conducted over the temperature range from −40 to 50 °C. Representative Nyquist plots measured at −40 °C are shown in Figure 2a,b. A single semicircle is observed in NaTaCl_6_ and samples with *x*_A_ = 0.1, 1.03, and 1.55 at the high-frequency region and can be fitted using an equivalent circuit comprising a parallel circuit of a constant-phase element (CPE) and a resistor. The corresponding capacitance values of those semicircles are 4.1 pF cm^–1^ (NaTaCl_6_), 4.5 pF cm^–1^ (*x*_A_ = 0.1), 6.3 pF cm^–1^ (*x*_A_ = 1.03), and 5.0 pF cm^–1^ (*x*_A_ = 1.55), which are consistent with bulk ion transport, as expected for the amorphous phase.^36^ For oxyhalide samples with other oxygen contents (0.21 ≤ *x*_A_ ≤ 0.88), no clear semicircle is observed even at −40 °C, suggesting much faster ion transport. The ionic resistances for those samples were extracted by fitting with the model by using the resistor to represent the bulk ion transport. The details can be found in the Experimental Section of the Supporting Information. The representative evaluation of electronic conductivity is also provided in Figure S4.

**Figure 2:**
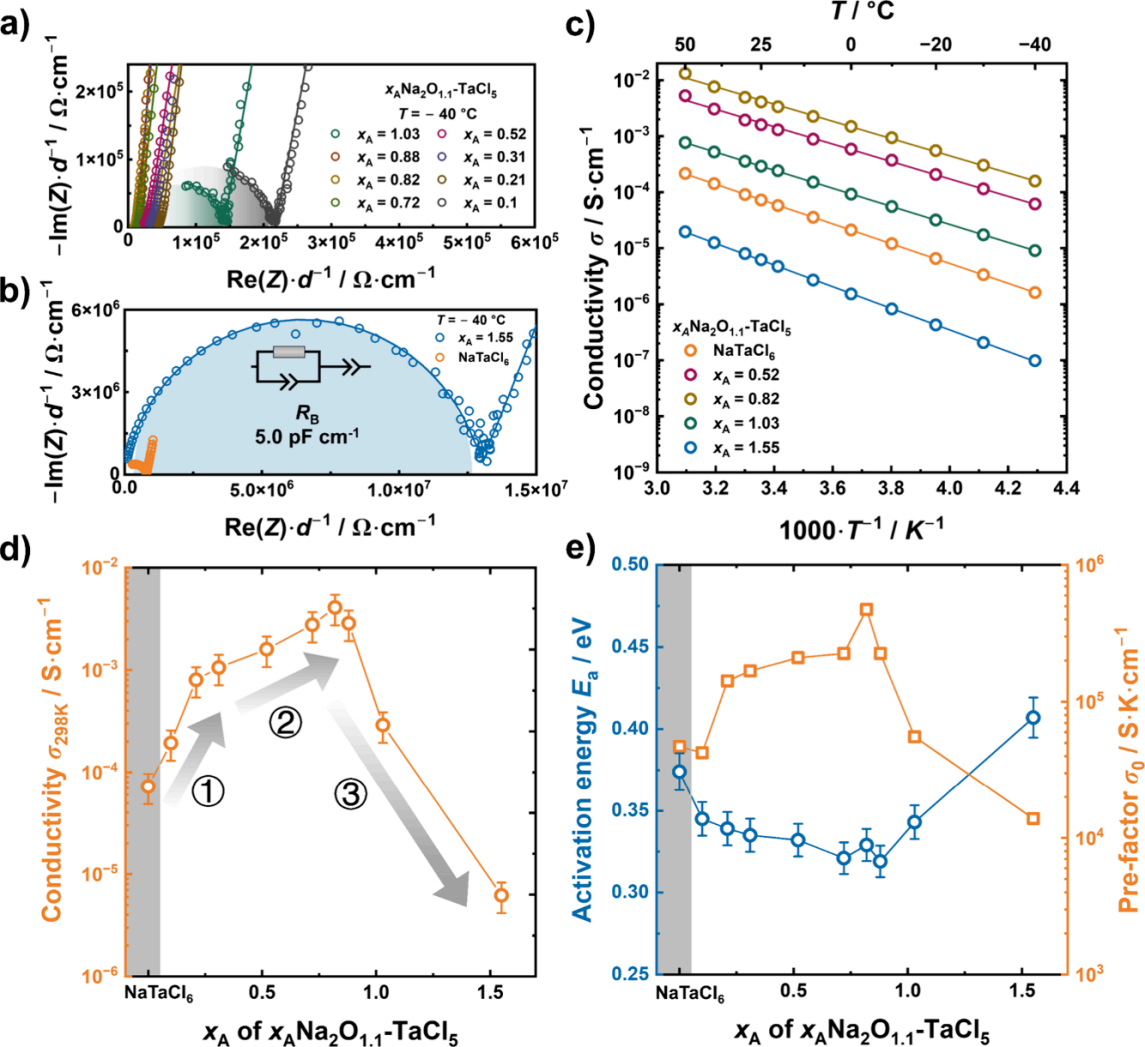
(a, b) Representative Nyquist plots measured at −40 °C, single semicircles attributable to bulk ion transport, with extracted capacitance values consistent with those expected for amorphous phases. (c) Arrhenius plots for representative samples, derived from ionic resistance obtained by EIS fitting. (d) Room-temperature (298 K) ionic conductivity and (e) corresponding activation energies and pre-exponential factors extracted from the Arrhenius relation. Error bars represent ±33% uncertainty in ionic conductivity and ±3% uncertainty in activation energy, which are referenced from our previous work on the reproducibility of ball-milled NaTaCl_6_.^35^

Arrhenius plots for representative *x*_A_Na_2_O_1.1_-TaCl_5_ samples are displayed in Figure 2c (full data sets in Figure S5), with corresponding room-temperature ionic conductivities (at 298 K) summarized in Figure 2d. The conductivity trends can be categorized into three compositional regions. Remarkably, even a trace incorporation of oxygen (*x*_A_ = 0.1) yields a conductivity higher than that of the monoanionic NaTaCl_6_. A sharp increase is observed up to *x*_A_ = 0.21 (region 1), followed by a moderate but steady rise to *x*_A_ = 0.82 (region 2), where the conductivity reaches 4.1 mS cm^–1^. This value exceeds previous reports on ball-milled analogues and is comparable to that of melt-quenched glasses.^19,26^ Beyond this composition, further oxygen incorporation leads to a pronounced drop in the conductivity (region 3).

To further elucidate the observed conductivity evolution, the activation energy (*E*_a_) and prefactor (σ_0_) (see Table S3) were extracted from the Arrhenius relation:

$$\sigma T=\sigma_{0}\;\exp\left(-\frac{E_{a}}{k_{B}T}\right)$$ 1

The prefactor σ_0_ can further be deconvoluted into components as follows:

$$\begin{array}{c} \sigma_{0}=\frac{\gamma n\nu a^{2}e^{2}}{k_{B}}\;\exp\left(\frac{\Delta S}{k_{B}}\right) \end{array}$$ 2

where γ is the geometrical factor, *n* is the carrier concentration, ν is the attempt frequency, *a* is the hopping distance, Δ*S* is the migration entropy, *e* is the electron charge, and *k*_B_ is the Boltzmann constant. The enhanced conductivity at *x*_A_ = 0.1 compared to NaTaCl_6_ is primarily due to a lower activation energy. Notably, although the carrier concentration (*n*) in *x*_A_ = 0.1 is nominally one-fifth that of NaTaCl_6_ (Na_0.2_TaO_0.1_Cl_6_), the comparable σ_0_ suggests significant enhancement in other contributing factors, likely related to vibrational properties (ν or Δ*S*) stemming from a highly disordered local structure. While the activation energy remains unchanged, σ_0_ increases sharply up to *x*_A_ = 0.21, accounting for the conductivity improvement in region 1. In region 2, the slope of the σ_0_ increase is moderate, and the trend appears to be dominated by an increase in the carrier concentrations. Theoretical scaling suggests a nearly linear dependence, with a 3.3-fold σ_0_ increase accompanying a 3.9-fold rise in Na content in nominal composition. At *x*_A_ = 0.82, the system exhibits a reduced *E*_a_ (0.32 eV) and more than an order-of-magnitude increase in σ_0_, resulting in the peak conductivity of 4.1 mS cm^–1^. Beyond this point, further oxygen incorporation leads to a sharp decline in σ_0_ and an increase in the migration barriers to 0.42 eV, contributing to the monotonic decrease in the conductivity observed in region 3. Although RIR analysis revealed that some insulating side phases exist in all samples, the estimated volume fractions of the amorphous phase in the samples remain higher than 70 vol %, even with *x*_A_ = 1.03 (see Table S4 and description in the Supporting Information). This level of side phase fractions is insufficient to account for the nearly order-of-magnitude drop in ionic conductivity based on the effective medium approximation (see the description in the Supporting Information). Therefore, the observed drop in ionic conductivity is attributable to the compositional change in the glass matrix. This section has demonstrated compositional evolution using XRD and Raman spectroscopy and has investigated ion transport behavior via temperature-dependent EIS. The observed conductivity trends, characterized by an initial sharp rise, a gradual increase, and a subsequent decline, correlate with compositional changes and phase evolution. These findings suggest that not only the overall amorphous nature but also subtle structural modifications play a key role in determining ion transport properties. Although trends in conductivity correlate with compositional changes, especially in oxygen and sodium content, establishing a direct link between specific structural motifs and ion transport remains an open challenge. Further structural investigation is needed to gain a deeper mechanistic understanding.

### Evolution of the Local Structural Change with Oxygen Incorporation

To uncover the underlying mechanisms governing ion transport, it is essential to investigate the local structure beyond macroscopic phase information. In the following section, we focus on elucidating the oxygen-mediated local coordination environments using advanced total scattering analysis and atomistic simulations. Synchrotron X-ray total scattering measurements were performed for representative samples: NaTaCl_6_ and *x*_A_Na_2_O_1.1_-TaCl_5_ (*x*_A_ = 0.51, 0.82, 1.03, and 1.55). The scattering vector *Q*_max_ reached ∼21.98 Å^–1^. The corresponding X-ray scattering patterns in *Q* and their structure factors are presented in Figure S6. All *x*_A_Na_2_O_1.1_-TaCl_5_ samples exhibit broad diffraction halos, indicating an amorphous nature, as expected. Pair distribution function (PDF) analysis was subsequently conducted, and the resulting *G*(*r*) functions are shown in Figure 3a. Whereas the PDF of NaTaCl_6_ exhibits broad but distinct peaks in the range of 6–7 Å, which originate from the Ta–Ta distance between two isolated TaCl_6_^–^ octahedra (red ticks in Figure 3a), the intensity of these peaks markedly decreases upon oxygen incorporation, indicating the disruption of long-range order among TaCl_6_^–^ units and consistent with the formation of an amorphous network.

**Figure 3:**
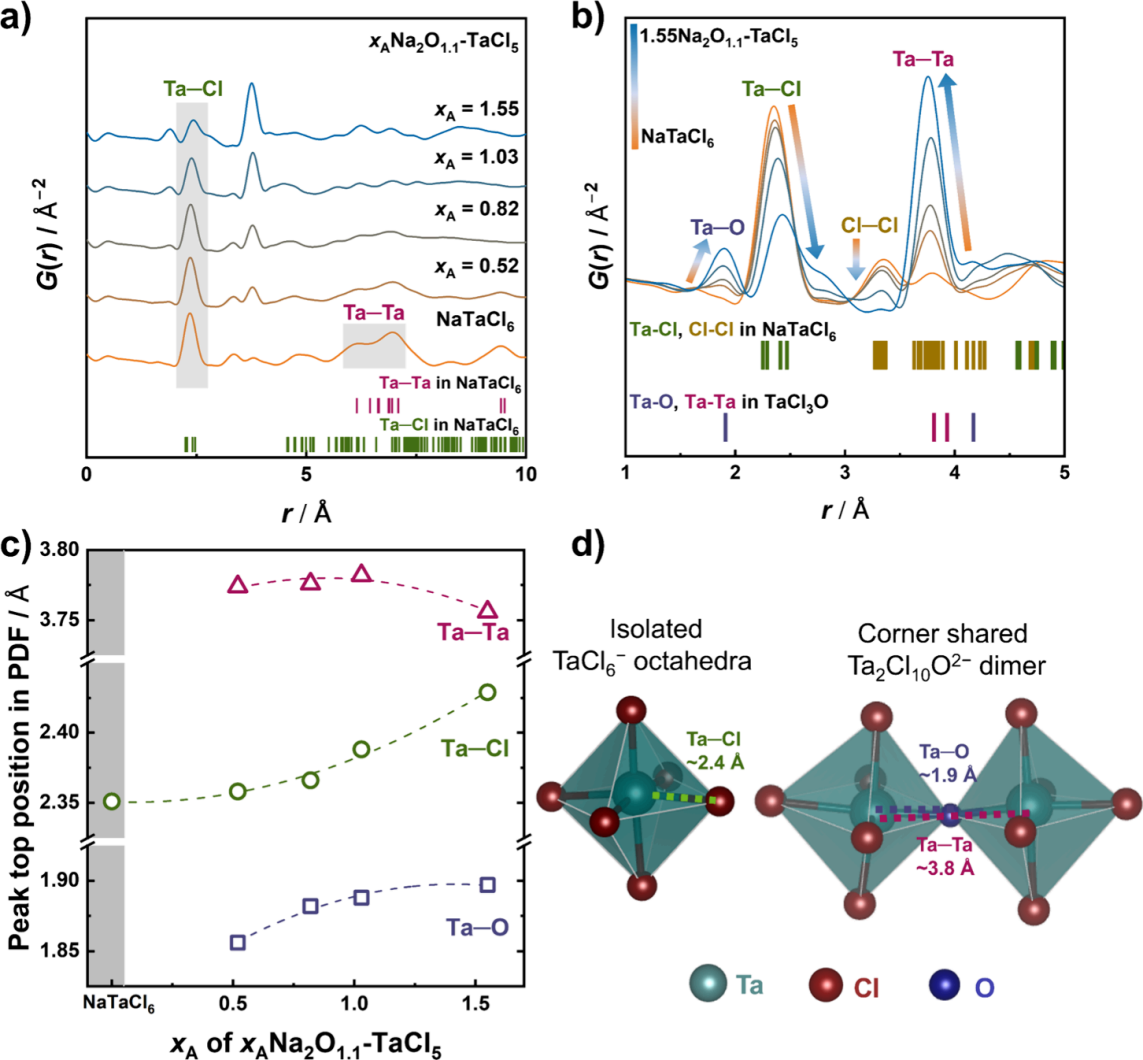
(a, b) Pair distribution function profiles obtained from synchrotron X-ray total scattering data. Tick marks indicate characteristic interatomic distances based on the crystal structures of NaTaCl_6_ and TaCl_3_O. (c) Summary of peak positions corresponding to Ta–Cl, Ta–Ta, and Ta–O bond lengths as derived from PDF analysis. (d) Schematic illustration of local atomic environments, highlighting representative Ta–Cl and Ta–O distances.

Figure 3b highlights the short-range structure (*r* ≤ 5 Å) through an overlay of magnified *G*(*r*) profiles. In NaTaCl_6_, a strong peak at ∼2.4 Å corresponds to the Ta–Cl bond in the TaCl_6_^–^ octahedra. This peak remains visible in the oxyhalides, but its intensity gradually reduces with an increasing oxygen content. The additional weaker peak at around 3.4 Å is assigned to Cl–Cl, consistent with the Cl–Cl distance in the NaTaCl_6_ structure. The absence of other peaks in NaTaCl_6_ is due to the lower electron density of Na compared to those of Ta and Cl. With an increasing oxygen content, new peaks emerge at ∼1.9 and ∼3.8 Å in oxychloride samples, which are absent in the NaTaCl_6_ structure. The ∼1.9 Å peak is consistent with Ta–O bond lengths observed in known tantalum oxides, such as LiTaO_3_ (1.89 Å) and Ta_2_O_5_ (1.95 Å), and is thus attributed to the formation of Ta–O bonds (purple ticks in Figure 3b). The ∼3.8 Å peak appears concurrently and is nearly twice the Ta–O bond length. This suggests its origin in Ta–Ta distances across bridging oxygen atoms (i.e., Ta–O–Ta linkages), as found in corner-sharing polyhedra such as the TaCl_10_O_2_^2–^ dimer (see Figure 3d). Similar trends were confirmed by laboratory X-ray total scattering (Ag Kα) on samples with *x*_A_ ≤ 0.31 (Figure S7). These results clearly indicate that oxygen incorporation leads to the substitution of Cl ligands with O, transforming isolated TaCl_6_^–^ units into a network of Ta–O–Ta linkages. This structural reorganization enhances amorphization and alters the ion transport pathways. Interestingly, the intensity of the Cl–Cl peak remains virtually unchanged up to *x*_A_ = 0.82 and then drops significantly, indicating a loss of Cl sitting next to another Cl atom bonded to Ta.

To gain further insight into local structural evolution, the shifts in peak positions of key atom pairs were analyzed (Figure 3c). With an increasing oxygen content, both the Ta–Cl and Ta–O bond distances show a general trend of elongation, whereas the Ta–Ta distances remain largely unchanged. This suggests a possible variation in the Ta–O–Ta bond angle, the extent of polyhedral connectivity, and/or the arrangement of multiple oxygen atoms within polyhedra as a structural response to oxygen incorporation, reflecting the increasing complexity of the local structure. In NaTaCl_6_, Ta^5+^ is known to be displaced from the center of the TaCl_6_^–^ octahedron due to the second-order Jahn–Teller effect, contributing to structural stability.^19,37^ In the amorphous oxychloride samples, the incorporation of oxygen appears to further enhance this off-center displacement of Ta^5+^ within its coordination environment. To exclude the influence of NaCl impurities, PDF data for NaCl were also collected. The positions of the newly observed peaks differ from those of interatomic distances in NaCl (Figure S8), confirming that the observed structural changes arise from the amorphous oxychloride matrix. Thus, the observed variations in peak intensity and position are attributed to local structural reorganization induced by the incorporation of oxygen incorporation. The trends in the local structure, as observed by Raman spectroscopy and PDF analysis, as well as those in ion transport as a function of oxygen content, were similarly examined in samples synthesized using Na_2_O_2_ instead of Na_2_O (see Figures S9–S11 and Table S5). In all cases, structural evolution and transport properties followed the same trends as those observed in Na_2_O-derived samples, and no significant differences in maximum ionic conductivity were found. These results confirm that the correlation between the local structure and ion transport properties in these oxyhalide glasses is primarily governed by oxygen content, regardless of the precursor. However, the exact oxygen content in Na_2_O_2_-derived samples may deviate from the nominal composition due to gas evolution upon prolonged ball milling; thus, a more detailed comparison with Na_2_O-derived samples requires further study.

To experimentally gain further insights into the exact position of the incorporated oxygen, X-ray absorption spectroscopy (XAS) and X-ray photoelectron spectroscopy (XPS) were performed (Figures S12 and S13). While the O K-edge XANES region has been reported to differentiate oxygen in different local bonding configurations in sodium borosilicate glasses,^38^ no apparent evolution in peak shape or position was monitored. In contrast, the O 1s XPS spectra in *x*_A_Na_2_O_1.1_-TaCl_5_ (*x*_A_ = 0.52, 0.82, 1.03, and 1.55) vary markedly with the oxygen content, indicating a change in the coordination environments of oxygen. However, the further assignment of the spectra remains challenging. No apparent changes were observed in Na 1s, Ta 4f, and Cl 2p spectra for NaTaCl_6_ and *x*_A_Na_2_O_1.1_-TaCl_5_ (*x*_A_ = 0.52, 0.82, 1.03, and 1.55).

Despite the experimentally observed changes in the local structure with increasing oxygen contents, the detailed coordination environments of the anion sublattice in amorphous oxychlorides remain elusive.

To further clarify local structural features, the local structures of *x*_A_Na_2_O-TaCl_5_ (*x*_A_ = 0.5, 0.8, 1.0, and 1.5) were investigated with *ab initio* molecular dynamics (AIMD) simulations (see the Supporting Information for details). Amorphous structures of *x*_A_Na_2_O-TaCl_5_ (*x*_A_ = 0.5, 0.8, 1.0, and 1.5) were generated using the standard melt-quench process simulated with *ab initio* molecular dynamics (AIMD). The melt-quench approach replicates the rapid cooling in nonequilibrium “melt-quench” synthesis of glass phases. The generated structures were then relaxed with density functional theory (DFT). At each composition, 10 isochronal AIMD snapshots were selected to statistically represent the amorphous structure and thereafter relaxed to 0 K with DFT to simulate quenching. Structures are shown in Figures S14–S17.

To validate the simulated structures, the corresponding pair distribution functions (PDFs) were computed and compared with the experimental data (Figure S18). The interatomic distances for Ta–O, Ta–Cl, and Ta–Ta generally reproduce the trends observed experimentally, although slight deviations in Ta–Ta distances are noted, likely due to limitations of the employed DFT functional. While experimental resolution limits direct comparison of Ta–O peaks, the consistent trends in Ta–Cl and Ta–Ta distances support the validity of the simulated amorphous structures. Nevertheless, it should be emphasized that the exact atomic configurations in amorphous materials can vary upon cooling and the simulated structure may differ from those realized experimentally, especially given that the present samples were synthesized via ball milling rather than melt-quenching. Thus, the simulated structures are interpreted as qualitative models.

Two key structural features emerge from the simulations:

(1) Oxygen coordination environments
  The incorporated oxygen atoms adopt two distinct coordination types, as illustrated in Figure 4a. One type connects two or more Ta-centered polyhedra to form Ta–O–Ta linkages and is referred to as bridging oxygen (BO). The other type coordinates to a single Ta atom and resides at a polyhedral corner, denoted as nonbridging oxygen (NBO). The compositional dependence of the BO/NBO ratio is shown in Figure 4b, with values averaged over 10 simulations per composition. At *x*_A_ = 0.5, ∼85% of the oxygen atoms are in BO configurations. However, as the oxygen content increases, the BO fraction decreases to ∼35% at *x* = 1.0 and remains roughly constant thereafter (the exact numbers are tabulated in Table S6). This trend suggests that oxygen preferentially occupies bridging sites at low concentrations, consistent with experimental PDF results shown in Figure 3b and Figure S7b in the Supporting Information, where Ta–Ta correlations emerge even at *x*_A_ = 0.1. The early onset of these Ta–Ta correlations likely reflects the dominant formation of BO species. This preferential BO formation offers a plausible explanation for why moderate oxygen incorporation does not hinder ion transport. Bridging oxygens are embedded within extended polyhedral frameworks and do not hinder Na-ion migration. In contrast, at higher oxygen contents, NBO species become more prevalent, occupying sites exposed to the surrounding free volumes and potentially interacting with mobile Na ions, thereby impeding their transport.^39,40^ As a result, the growing population of NBO increasingly obstructs ion transport pathways, resulting in a sharp drop in the prefactor and, ultimately, an abrupt increase in the activation energy, as seen in region 3 in Figure 2.
(2) Na-ion coordination and free volume
  Oxygen incorporation promotes the formation of the amorphous phase, resulting in significant changes to the Na-ion coordination environment (Figure 4c). Compared with crystalline NaTaCl_6_, the amorphous oxychlorides at *x*_A_ = 0.5 exhibit a markedly lower average Na^+^ coordination number (CN) and increased Voronoi volume. The Voronoi volume of Na^+^ ion is defined as the region of space that is closer to that Na^+^ ion than to any other ion in the system.^41,42^ The Voronoi volume is commonly employed as a proxy for the free volume, and in this work, it is used to characterize the local free volume of Na^+^ ions in the amorphous oxychlorides. The CN decreases up to *x*_A_ = 0.8 and stabilizes thereafter, while the free volume peaks at *x*_A_ = 0.5 and decreases monotonically with further oxygen addition, eventually falling below that of the crystalline counterpart at *x*_A_ ≥ 1.0. A large free volume is commonly observed in ion conducting glasses and is known to facilitate ion transport by providing wider bottleneck sizes and a greater number of accessible sites for ion migration.^39,43–45^

**Figure 4:**
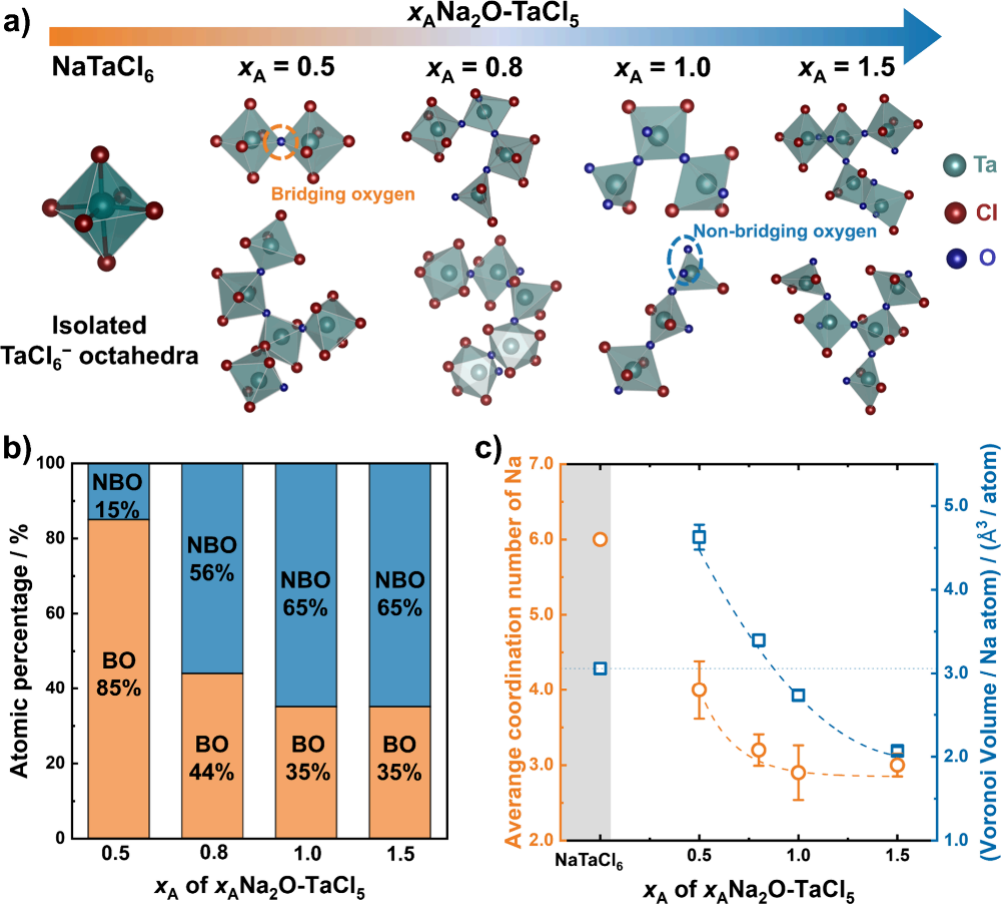
(a) Representative local structures obtained from *ab initio* molecular dynamics simulations of amorphous *x*_A_Na_2_O–TaCl_5_ compositions. (b) Atomic fractions of bridging and nonbridging oxygen species, illustrating the preferential occupation of oxygen at bridging sites at a low oxygen content. (c) Averaged Na-ion coordination number and Voronoi volume per Na atom, derived from the simulated structures.

These structural trends align well with the experimentally observed ionic conductivity. The steep conductivity enhancement at low oxygen contents (*x*_A_ ≤ 0.31) can be attributed to the onset of amorphization, leading to reduced Na CN and increased free volume. Additionally, the predominance of BO species at these compositions avoids the creation of electrostatic barriers along the Na-ion migration pathways. As the Na_2_O content increases, the concentration of mobile carriers and the degree of structural disorder continue to rise, reaching an optimal balance between the BO ratio, Na CN, and free volume within the range 0.52 ≤ *x*_A_ ≤ 0.85. Consequently, the ionic conductivity remains high in this region. Beyond this range, excessive oxygen incorporation leads to a reduction in the BO ratio and free volume, resulting in a decline in ionic conductivity at *x*_A_ = 1.03 and 1.55. While substitution in crystalline materials generally influences ionic conductivity through both global and local structural changes,^46^ ion transport in amorphous phases is primarily governed by local features, underscoring the importance of controlling the local structural environment.

## Conclusions

We have elucidated the role of oxygen incorporation in tuning the structure and ion transport properties of amorphous Na_2_O-TaCl_5_ oxyhalide glasses. Through a combination of electrochemical measurements, total scattering experiments, and *ab initio* molecular dynamics simulations, we demonstrated that moderate oxygen incorporation induces amorphization, reduces the Na-ion coordination number, and increases the local free volume, which are key features that facilitate Na-ion mobility. Simulations further revealed a strong preference for bridging oxygen species at a low oxygen content, which form extended Ta–O–Ta networks without obstructing ion transport pathways. At higher oxygen concentrations, the increasing prevalence of nonbridging oxygen and the concurrent reduction in free volume correlate with a decline in conductivity. An optimal compositional window (*x*_A_ ≈ 0.5–0.85) was identified, in which ionic conductivities above 4 mS cm^–1^ were achieved. These findings highlight the importance of local anion coordination in governing ion dynamics and provide a structural framework for the design of multianion glass electrolytes for sodium-based solid-state batteries.

## Supplementary Material



## Notes

Description of experimental and computational methods; description of actual composition; EDS data; Rietveld results for RIR analysis; additional Raman spectra; DC polarization data; Arrhenius plots of all samples; estimation of volume fractions of the amorphous phase; synchrotron X-ray total scattering patterns and *S*(*Q*) data of NaTaCl_6_ and *x*_A_Na_2_O_1.1_-TaCl_5_ (*x*_A_ = 0.52, 0.82, 1.03, and 1.55); PDF analysis data of *x*_A_Na_2_O_1.1_-TaCl_5_ (*x*_A_ = 0.1, 0.21, and 0.31) and NaCl; samples synthesized using Na_2_O_2_ as the precursor; XPS and XAFS data; simulated local structure of *x*_A_Na_2_O-TaCl_5_ (*x*_A_ = 0.5, 0.8, 1.0, and 1.5) and corresponding PDF analysis results (PDF)
