# Supplementary material for: Oxygen-Mediated Structural Modulation and Ion Transport in xNa2O‑TaCl5 Glass Electrolytes
Source: J Am Chem Soc. 2025 Nov 11;147(47):43391–9. doi: 10.1021/jacs.5c10564 (PMC12673589; doi:10.1021/jacs.5c10564)
Supplement: Supplementary file 1 [file ja5c10564_si_001.pdf]

Supporting Information

Oxygen-Mediated Structural Modulation and Ion Transport in xNa$_2$O-TaCl$_5$ Glass Electrolytes

Zheng Huang,[a,b,c] Neha Yadav,[d] Shun Itakura,[a,b] Peng Song,[a] Hirofumi Akamatsu,[c] Katsuro Hayashi,[c] Prashun Gorai,[d,e*] Saneyuki Ohno[a,b,c*]

[a] *Institute of Multidisciplinary Research for Advanced Materials, Tohoku University, 2-1-1 Katahira, Aoba-ku, Sendai, Miyagi 980-8577, Japan*

[b] *Department of Frontier Sciences for Advanced Environment, Graduate School of Environmental Studies, Tohoku University, 2-1-1 Katahira, Aoba-ku, Sendai, Miyagi 980-8577, Japan*

[c] *Department of Applied Chemistry, Graduate School of Engineering, Kyushu University, 744 Motooka, Nishi-ku, Fukuoka 819-0395, Japan*

[d] *Department of Metallurgical and Materials Engineering, Colorado School of Mines, Golden, Colorado 80401, United States*

[e] *Chemical & Biological Engineering, Rensselaer Polytechnic Institute, Troy, New York 12180, United States*

**E-mail: saneyuki.ohno.c8@tohoku.ac.jp, goraip@rpi.edu*

Table of contents:

1. Experimental and Computational Methods

2. Relationship between nominal composition and actual composition

3. EDS data of samples

4. Rietveld analysis results of the sample mixed with Si for relative intensity ratio analysis

5. Additional Raman spectra

6. Evaluation of electronic conductivity

7. Arrhenius plots of all samples and values of activation energy and pre-factor

8. Estimation of volume fractions and ionic conductivity of amorphous phase

9. Synchrotron X-ray total scattering patterns and $S(Q)$ data of NaTaCl$_6$ and x_ANa$_2$O$_{1.1}$-TaCl$_5$ (x_A = 0.52, 0.82, 1.03, and 1.55)

10. PDF analysis data of x_ANa$_2$O$_{1.1}$-TaCl$_5$ (x_A = 0.1, 0.21, 0.31) and NaCl

11. XRD, Raman spectroscopy, PDF analysis, and ion transport properties of samples synthesized using Na_2O_2 as precursor

12. XPS and XAFS of $NaTaCl_6$ and $x_A Na_2O_{1.1}$-$TaCl_5$ (x_A = 0.52 and 1.03)

14. Simulated local structure of $x_A Na_2O$-$TaCl_5$ (x_A = 0.5, 0.8, 1.0, and 1.5), table of bridging and non-bridging oxygen, and corresponding PDF analysis results

1. Experimental Section

Synthesis

A stoichiometric mixture of raw materials NaCl (99%, Sigma-Aldrich), Na_2O (80%, Sigma-Aldrich), and $TaCl_5$ (99.99%, Sigma-Aldrich) were filled into ZrO_2 cups with a 30:1 mass ratio for 3 mm ZrO_2 balls under an Ar atmosphere. The ball milling was conducted using PULVERISETTE 7 (Fritsch GmbH) with the setting of 500 rpm for 600 cycles (10 min milling and 10 min pause for each cycle). For comparison, $NaTaCl_6$ was also synthesized with the same milling duration. As reported in our prior work,[1] we compared samples of $NaTaCl_6$ synthesized at different milling durations (99 vs. 600 cycles). While longer milling slightly improved ionic conductivity, it did not result in substantial changes in crystallinity or phase composition. These results indicate that prolonged milling alone is not sufficient to induce amorphization. Therefore, we believe that the significant amorphization observed in the oxygen-containing samples arises primarily from oxygen incorporation itself, which destabilizes the long-range order of the halide lattice and promotes glass formation. In this study, the choice of 600 cycles was made to ensure compositional homogeneity across all samples and facilitate a meaningful comparison of oxygen's structural role.

Sample Characterization

Powder X-ray diffraction: The powder X-ray diffraction was measured by D8 ADVANCE (Bruker AXS GmbH, Karlsruhe, Germany) with Cu Kα radiation. To avoid sample powder exposure to air during measurement, samples were sealed into an airtight holder with Cu film cover.

Relative Intensity Ration analysis: To assess the amorphous phase fraction of oxyhalide samples, the relative intensity ratio analysis was conducted. The sample powder was hand-mixed with crystalline Si with a fixed weight ratio of *m*(Si), which was 10 wt% in this study, and the X-ray diffraction data of the mixture was collected for the subsequent Rietveld refinement. By obtaining the "apparent" weight ratio of Si, *m*'(Si), and crystalline side phase(s) of *i*, *m*'(side phase, *i*), the "actual" phase fractions of side phases *m*(side phase, *i*) and amorphous content *m*(amorphous) can be then quantified from the following relations:

$$m(\text{amorphous}) + \sum_{i=1}^{n} m(\text{side phase}, i) \; + m(\text{Si}) = 100\ \%, \quad (1)$$

where

$$m(\text{side phase}, i) = m'(\text{side phase}) \cdot \frac{m(\text{Si})}{m'(\text{Si})}. \quad (2)$$

Total scattering: The synchrotron X-ray total scattering was measured using the beamline BL13XU at Spring8 (Japan). The powder samples were sealed in a Lindemann glass capillary tube (inner diameter 0.3 mm) under Ar atmosphere. The wavelength of the beam was determined to be 0.3543 Å by a

calibration with CeO_2 standard sample. The measurement was carried out by scanning 2θ angles from 0.60° to 76.51°, which corresponds to a scattering vector Q range from 0.19 Å^{-1} to 21.98 Å^{-1}.
The lab X-ray total scattering data for pair distribution function analysis was measured by Empyrean (Malvern Panalytical, United Kingdom) with Ag Kα radiation. The powder samples were sealed in a Lindemann glass capillary tube (inner diameter 0.3 mm) under Ar atmosphere. The measurement was carried out by scanning 2θ angles from 3.01° to 143.84°, which corresponds to a scattering vector Q range from 0.60 Å^{-1} to 21.28 Å^{-1}.

Structure analysis: The crystal structure was visualized via VESTA software package.[2] The Rietveld refinement of the obtained reflection patterns was conducted via TOPAS-Academic V6 software.[3]

Pair distribution function analysis: The pair distribution function analysis was carried out using software based on Igor Pro (http://rud.spring8.or.jp/member/0020758/distrbution.html). The pair distribution function $G(r)$ was obtained by conducting a Fourier transform on the structure factor $S(Q)$ of XRD data with a window function developed by Lorch.[4,5] The scattering vector Q was chosen to ~21.98 Å^{-1} for synchrotron X-ray total scattering data, and ~21.28 Å^{-1} for lab Ag-tube X-ray total scattering data.

Raman analysis: Raman spectra were measured by LabRAM Aramis (HORIBA Ltd., Japan) and inVia Raman microscope (Renishaw plc, United Kingdom) using the laser with 532 nm wavelength. The measurement was conducted with a 50x objective lens and an 1800 l/mm diffraction grating. The laser power and integration time were optimized to prevent damage to the sample. To avoid sample powder exposure to air during measurement, samples were sealed between two glass slides using vacuum grease under Ar atmosphere.

X-ray absorption spectroscopy: X-ray absorption spectroscopy was measured using the beamline BL08U at NanoTerasu (Japan). Sample powder was fixed on carbon tape in an appropriate amount. A designed transfer vessel was used to transfer the samples from glove box to the instrument without air exposure. The spectra of O *K*-edge were recorded in total electron yield mode. Spectra were collected from 520 eV to 560 eV with a step size of 0.5 eV.

X-ray photoelectron spectroscopy: X-ray photoelectron spectroscopy was measured using PHI5000 VersaProbe II (ULVAC-PHI Ltd., Japan) with a monochromatic Al Kα source (hv = 1486.6 eV). An airtight transfer vessel was employed to prevent air exposure during sample transfer. A X-ray powder of 25 W was used. A pass energy of 117.4 eV was utilized for survey scans and 58.7 eV was utilized for detailed spectra. The obtained binding energy scale was calibrated with C 1s peak to the bonding energy of 284.6 eV. Data analysis was carried out using CasaXPS software.

Energy dispersive X-ray spectroscopy: Energy dispersive X-ray spectroscopy was measured using JSM-7800F (JEOL Ltd., Japan) and Inca x-act (Oxford Instruments, UK). Samples were measured in pellet form, and an airtight transfer vessel was employed to prevent air exposure during transfer. To characterize the composition, four regions were selected, and five points were analyzed in each region.

Electrochemical impedance spectroscopy: The ion transport properties of synthesized samples were evaluated by temperature-dependent electrochemical impedance spectroscopy with a home-made air-tight cell. Roughly 200mg of sample powder was filled into the press cell, pressed by two cylindrical stainless bars. After the cell was constructed, a uniaxial pressure of 300 MPa was applied to stainless bars for 3 minutes. The stainless-steel frame held a ca. 60 MPa pressure during measurement.

The electrochemical measurements were performed on VMP3 impedance analyzer (Biologics) using a 20 mV voltage amplitude with frequency from 1 MHz to 10 mHz. The temperature range was settled from – 40 to 50 °C. The cell was placed in a climate chamber to control the cell temperature during testing. The Relaxis3 software was used to fit the obtained impedance data.

For Nyquist plots where a single semicircle is observed in the high-frequency region, the impedance spectra were fitted using an equivalent circuit comprising a resistor in parallel with a constant phase element (CPE), connected in series with another CPE. For Nyquist plots showing no clear semicircle, a simplified circuit model comprising a resistor in series with a CPE was used. However, in cases where the blocking tail could not be well fitted by a single CPE, an additional parallel combination of a resistor and a CPE was inserted between the resistor and the tail CPE. The capacitance associated with this element (~ 10^{-7} F cm^{-1}) suggests it originates from the interface between the stainless-steel bars and the sample and is not related to the sample's bulk resistance.

Direct-current polarization measurement: The electronic conductivity of synthesized samples was evaluated by direct-current polarization measurement. Roughly 120mg of sample powder was filled into the press cell, pressed by two cylindrical stainless bars. After the cell was constructed, a uniaxial pressure of 300 MPa was applied to stainless bars for 3 minutes. The stainless-steel frame held a ca. 60 MPa pressure during measurement. The measurements were performed on VMP3 impedance analyzer (Biologics) with applied voltage of 100 mV, – 200 mV, and 250 mV. The temperature was maintained at 25 °C during measurement.

Computational

Amorphous structures of $x_A Na_2O$-$TaCl_5$ (x_A = 0.5, 0.8, 1.0, and 1.5) were generated using the standard melt-quench process simulated with *ab initio* molecular dynamics (AIMD). The melt-quench approach replicates the rapid cooling in non-equilibrium 'melt-quench' synthesis of glass phases. The generated structures were then relaxed with density functional theory (DFT).

The initial structures for AIMD simulations of $x_A Na_2O$-$TaCl_5$ (x = 0.5, 0.8, 1.0, and 1.5) were constructed by randomly arranging Na, Ta, Cl, and O atoms corresponding to the different compositions in a cubic

simulation box using the PACKMOL software package.[6] The volumes of the cubic simulation boxes were set to 20% larger than the volume of the corresponding $NaTaCl_6$ (crystalline), which ensures a minimum distance of 2 Å between the atoms in the initial arrangements. These geometric constraints ensure that the random atomic arrangements to facilitate equilibration in AIMD simulations and to prevent the formation of unphysical, high-energy structures. Subsequently, AIMD simulations were performed on the initial configurations within the canonical (NVT) ensemble, maintain a temperature of 2000 K using the Noose-Hoover thermostat. The temperature was selected as approximately 500 K above the melting points of the constituents, to ensure rapid equilibration of the disordered phase. Each simulation comprised 5000 steps for equilibration followed by 5000 production steps, with a time step of 2 femtoseconds. From the production run of the AIMD simulation, 10 independent 'isochronal' snapshots were selected to statistically represent the amorphous structure and thereafter, relaxed to 0 K with DFT to simulate quenching. Statistical convergence of the AIMD melt-quench simulations was assessed by monitoring temperature stabilization in the NVT ensemble. Also, Pair Distribution Functions (PDFs) computed for the 10 isochronal snapshots showed negligible variation, confirming that the structural descriptors are well converged.

Both AIMD and DFT relaxations were performed with the Vienna Ab Initio Simulation Package (VASP, version 5.4.4), employing the projector-augmented wave (PAW) method to treat core and valence electrons.[7–9] The exchange-correlation functional was described by the Perdew-Burke-Ernzerhof (PBE) formulation within the Generalized Gradient Approximation (GGA).[10] The PAW pseudopotentials used were: Na_pv 05Jan2001, Ta_pv 07Sep2000, Cl 17Jan2003 and O_s 07Sep2000. Dispersion corrections were not applied in either AIMD or DFT relaxations, as our preliminary tests revealed no qualitative difference in the amorphous structural models, particularly in the formation of bridging and non-bridging oxygens. Structural optimization was carried out until the forces on each atom were reduced to below 0.01 eV/Å, employing a plane-wave energy cutoff of 340 eV and a Gamma-centered ***k***-point grid determined by a length factor (R_k) of 20. The setup and analysis of the calculations were performed using the PyLada framework.[11] The relaxed structures were visualized with VESTA.[2]

Pair Distribution Function (PDF) analysis was carried out for four compositions of amorphous $x_A Na_2O$-$TaCl_5$, with $x_A = 0.5$, 0.8, 1.0, and 1.5. At each composition, 10 DFT-relaxed structures (from isochronal snapshots) were used, and the resulting data were averaged to obtain representative structure functions. The partial pair distribution functions, *g(r)*, were computed using the OVITO software.[12] These *g(r)* functions were then transformed into partial reduced pair distribution functions, *G(r)*, using custom Python scripts and tools from the PyLada package. The total *G(r)* were obtained by summing the partial *G(r)* values, following the Morningstar-Warren approximation to account for the appropriate weighting and correlations between atomic species.[13] In addition to the PDF analysis, Voronoi volumes were calculated using the Voronoi Analysis modifier in OVITO, and the results were averaged over the 10 structures per composition to ensure statistical reliability. The coordination number analysis was performed using PyLada.

2. Relationship between nominal composition and actual composition

Table S1 *The actual composition of samples when 10 mol% Na_2O_2 is considered.*

Nominal composition $x_NNa_2O_{1.2}$-$TaCl_5$	Actual composition $x_ANa_2O_{1.1}$-$TaCl_5$	Actual ratio of Na : Ta : Cl : O
0.1 $Na_2O_{1.2}$-$TaCl_5$	0.1 $Na_2O_{1.1}$-$TaCl_5$	0.2 : 1 : 5 : 0.11
0.2 $Na_2O_{1.2}$-$TaCl_5$	0.21 $Na_2O_{1.1}$-$TaCl_5$	0.42 : 1 : 5 : 0.231
0.3 $Na_2O_{1.2}$-$TaCl_5$	0.31 $Na_2O_{1.1}$-$TaCl_5$	0.62 : 1 : 5 : 0.341
0.5 $Na_2O_{1.2}$-$TaCl_5$	0.52 $Na_2O_{1.1}$-$TaCl_5$	1.04 : 1 : 5 : 0.572
0.7 $Na_2O_{1.2}$-$TaCl_5$	0.72 $Na_2O_{1.1}$-$TaCl_5$	1.44 : 1 : 5 : 0.792
0.8 $Na_2O_{1.2}$-$TaCl_5$	0.82 $Na_2O_{1.1}$-$TaCl_5$	1.64 : 1 : 5 : 0.902
0.85 $Na_2O_{1.2}$-$TaCl_5$	0.88 $Na_2O_{1.1}$-$TaCl_5$	1.76 : 1 : 5 : 0.968
1.0 $Na_2O_{1.2}$-$TaCl_5$	1.03 $Na_2O_{1.1}$-$TaCl_5$	2.06 : 1 : 5 : 1.133
1.5 $Na_2O_{1.2}$-$TaCl_5$	1.55 $Na_2O_{1.1}$-$TaCl_5$	3.10 : 1 : 5 : 1.705

Since the commercial Na_2O precursor is specified to contain 20 mol % Na_2O_2, and Na_2O_2 can also serve as a precursor for sodium oxyhalides,[14] we initially set our nominal composition to $x_NNa_2O_{1.2}$-$TaCl_5$ (x_N = 0.1, 0.2, 0.3, 0.5, 0.7, 0.8, 0.85, 1.0, and 1.5). However, the actual presence of c.a. 10 mol% Na_2O_2 in Na_2O precursor was confirmed by Rietveld refinement (see Figure 1a in the main text), thereby the actual sodium and oxygen contents of the synthesized samples slightly deviate from the nominal composition. The actual nominal composition was calculated as $x_ANa_2O_{1.1}$-$TaCl_5$ (x_A = 0.1, 0.21, 0.31, 0.52, 0.72, 0.82, 0.88, 1.03, and 1.55).

3. EDS data of $1.03Na_2O_{1.1}$-$TaCl_5$.

Table S2 *The atomic percentage of Na, Ta, Cl, O, and Zr of $1.03Na_2O_{1.1}$-$TaCl_5$ obtained from EDS and comparison with expected composition. Four regions were selected, and five points were analyzed in each region.*

	**Na at%**	**Ta at%**	**Cl at%**	**O at%**	**Zr at%**
**Measured**	19.1(9)	12.2(7)	42(1)	26(2)	0.01(1)
**Expected**	22.4	10.9	54.4	12.3	

4. Rietveld analysis results of the sample mixed with Si for relative intensity ratio analysis.

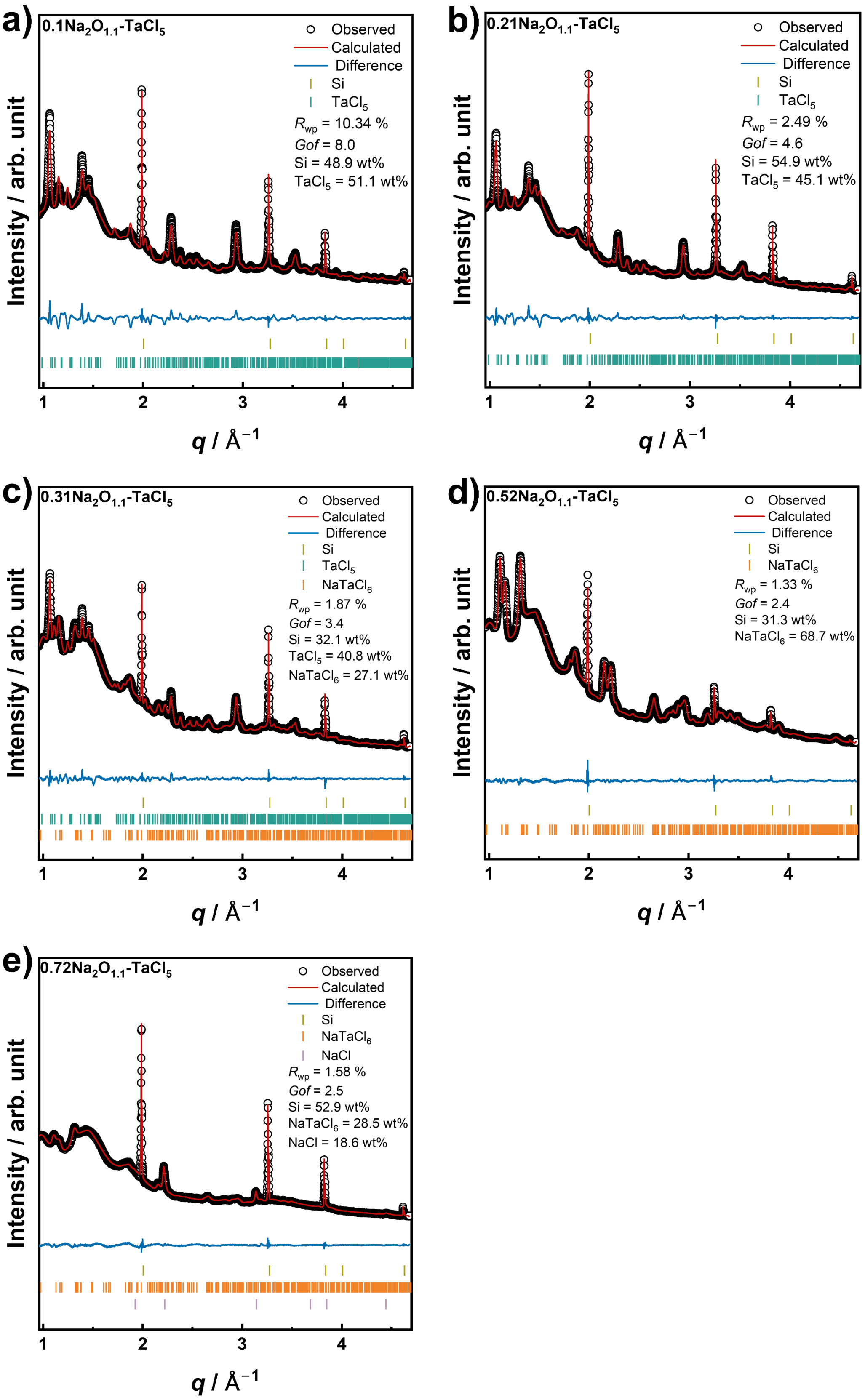

Figure S1 *Rietveld refinement results of $x_A Na_2O_{1.1}$-$TaCl_5$ (x_A = 0.1, 0.21, 0.31, 0.52, and 0.72) mixed with a fixed mass ratio of Si (10 wt%) for relative intensity ratio analysis.*

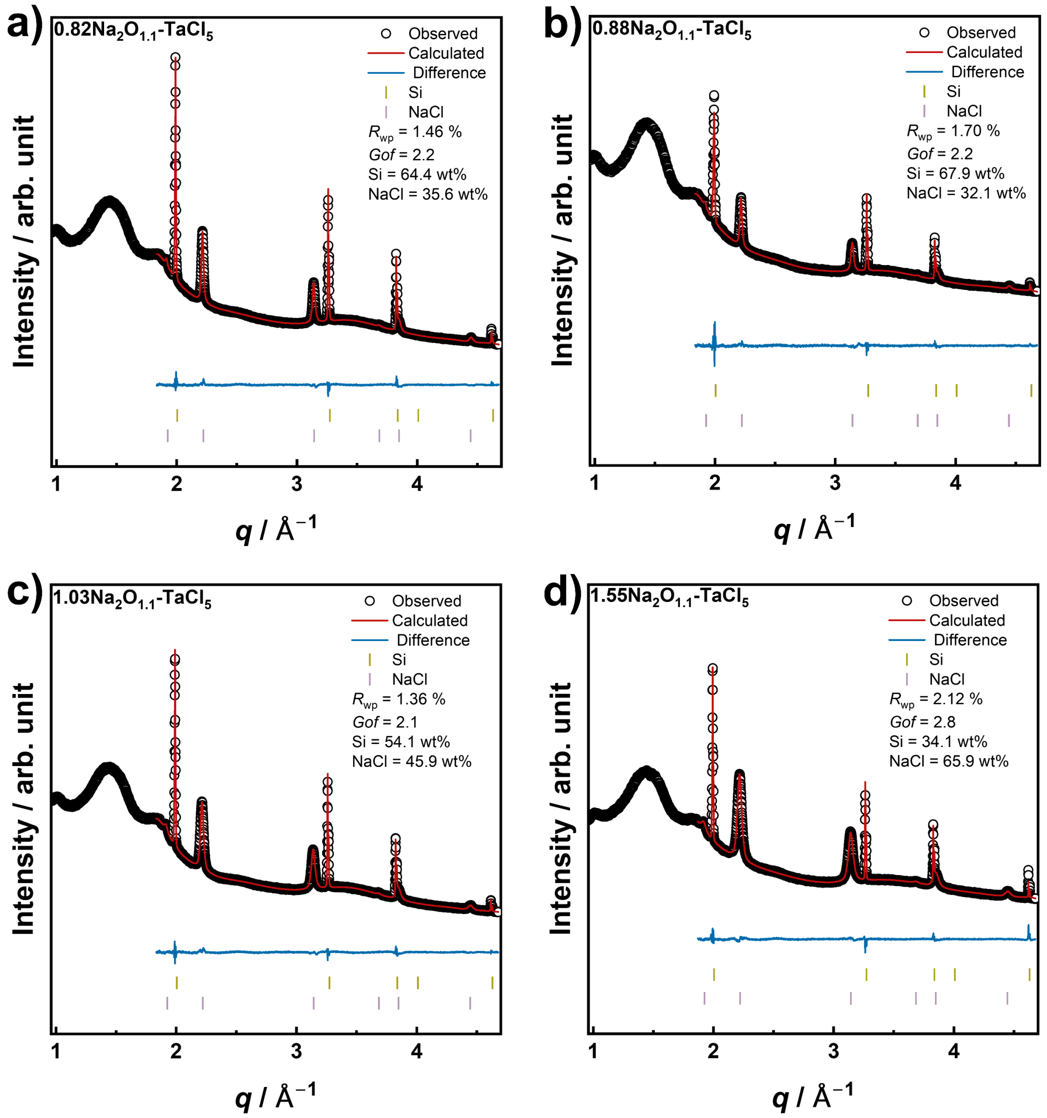

Figure S2 *Rietveld refinement results of* $x_A Na_2O_{1.1}$*-*$TaCl_5$ *(*x_A *= 0.82, 0.88, 1.03, and 1.55) mixed with a fixed mass ratio of Si (10 wt%) for relative intensity ratio analysis. The fitting range was set from q = 1.87* $Å^{-1}$*, below which no diffraction peaks are observable and no Bragg diffraction peaks of NaCl and Si are theoretically present.*

5. Additional Raman spectra

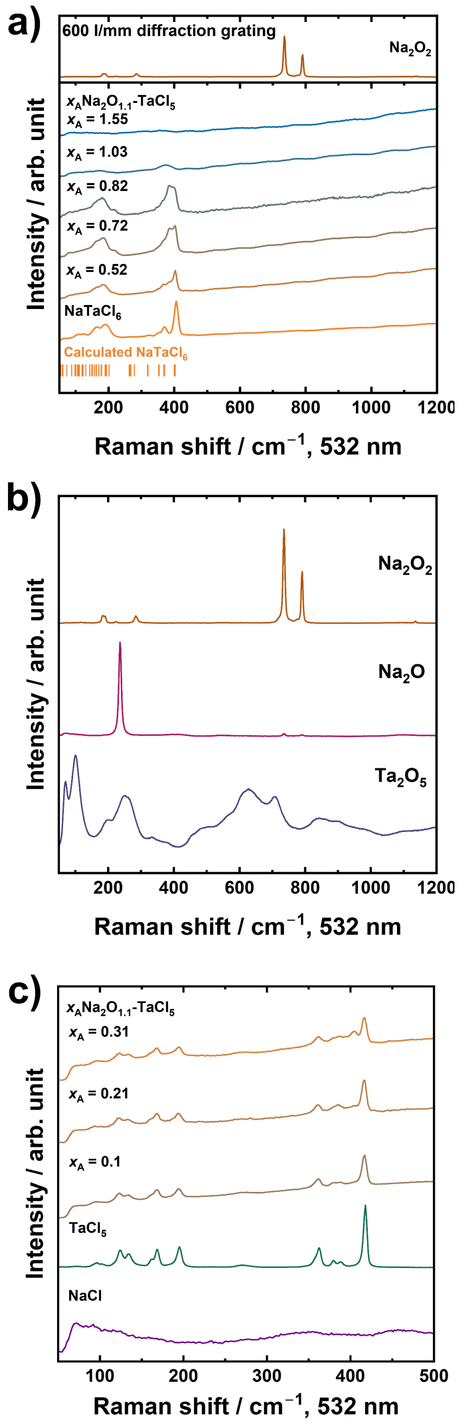

Figure S3 *(a)Raman spectra of $x_ANa_2O_{1.1}$-$TaCl_5$ (x_A = 0.52, 0.72, 0.82, 1.03, and 1.55) shown in the Raman shift range from 50 to 1200 cm^{-1}. To mitigate spectrograph-switching-induced spectral offset, measurements were repeated using a 600 l/mm^{-1} grating. No apparent Raman peaks assignable to O_2^{2-} ions or Ta–O vibrations were observed. (b) Raman spectra of Ta_2O_5, Na_2O, and Na_2O_2 as reference. (c)Raman spectra of $x_ANa_2O_{1.1}$-$TaCl_5$ (x_A = 0.1, 0.21, and 0.31), $TaCl_5$, and NaCl. The vibrational signal originating from $TaCl_5$ can be observed when x_A = 0.1, 0.21, and 0.31, and the vibrational signal originating from $NaTaCl_6$ can be observed in the sample with x_A = 0.31, consistent with the results of XRD.*

6. Evaluation of electronic conductivity

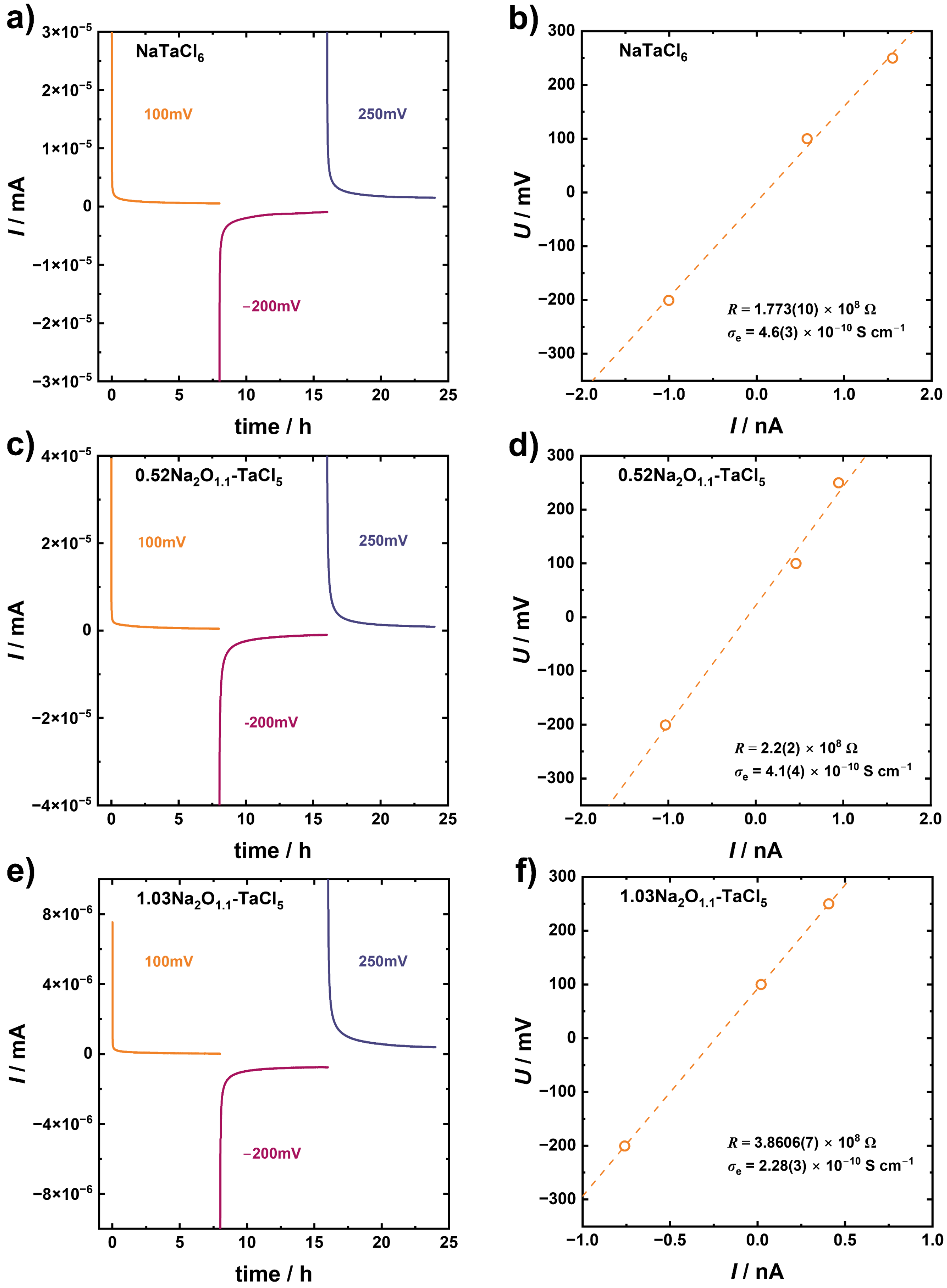

Figure S4 *Direct-current polarization curves of (a) $NaTaCl_6$, (c) $0.52Na_2O_{1.1}$-$TaCl_5$, and (e) $1.03Na_2O_{1.1}$-$TaCl_5$, with (b, d, and f) corresponding linear fits between applied voltages and stabilized currents.*

7. Arrhenius plots of all samples

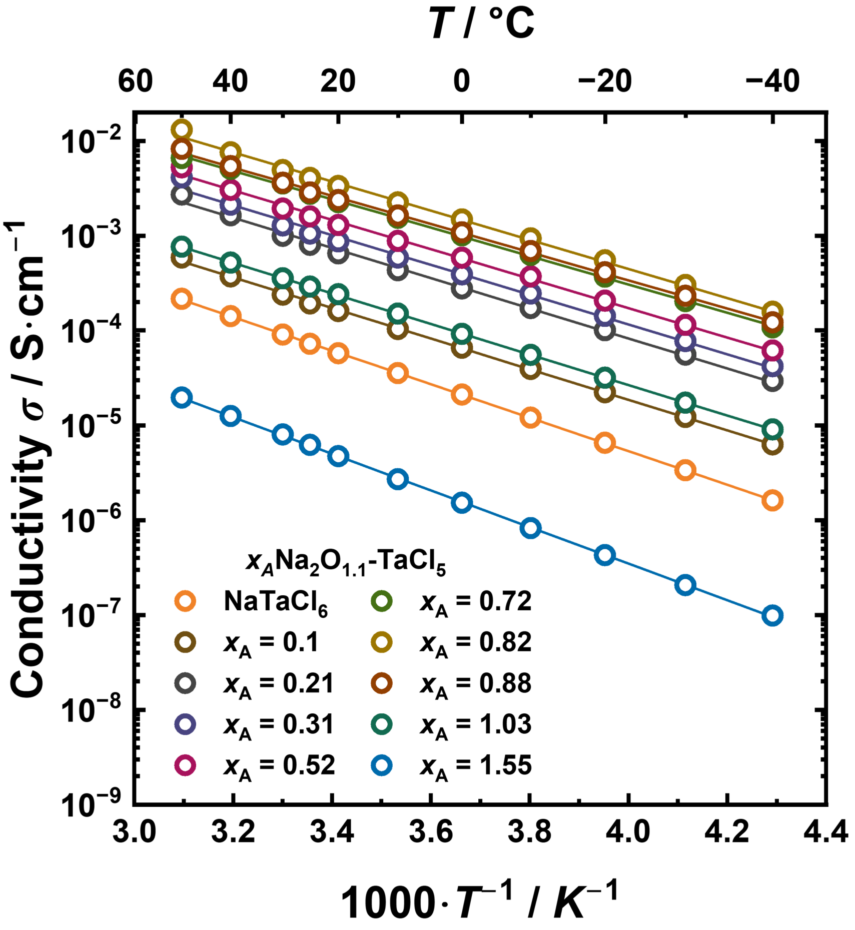

Figure S5 *Arrhenius plots of synthesized samples.*

Table S3 *The activation energy, pre-factor, and ionic conductivity of synthesized samples*

Actual chemical formula	Ionic conductivity at 298K / S cm^{-1}	Activation energy / eV	Pre-factor / S K cm^{-1}
$NaTaCl_6$	$7.2(24) \times 10^{-5}$	0.374(11)	4.70×10^4
0.1 $Na_2O_{1.1}$-$TaCl_5$	$1.9(6) \times 10^{-4}$	0.345(10)	4.23×10^4
0.21 $Na_2O_{1.1}$-$TaCl_5$	$8.0(26) \times 10^{-4}$	0.339(10)	1.42×10^5
0.31 $Na_2O_{1.1}$-$TaCl_5$	$1.1(4) \times 10^{-3}$	0.335(10)	1.68×10^5
0.52 $Na_2O_{1.1}$-$TaCl_5$	$1.6(5) \times 10^{-3}$	0.332(10)	2.10×10^5
0.72 $Na_2O_{1.1}$-$TaCl_5$	$2.8(9) \times 10^{-3}$	0.321(10)	2.25×10^5
0.82 $Na_2O_{1.1}$-$TaCl_5$	$4.1(14) \times 10^{-3}$	0.329(10)	4.74×10^5
0.88 $Na_2O_{1.1}$-$TaCl_5$	$2.9(10) \times 10^{-3}$	0.319(10)	2.25×10^5
1.03 $Na_2O_{1.1}$-$TaCl_5$	$2.9(10) \times 10^{-4}$	0.343(10)	5.54×10^4
1.55 $Na_2O_{1.1}$-$TaCl_5$	$6.2(20) \times 10^{-6}$	0.407(12)	1.39×10^4

8. Estimation of volume fractions and ionic conductivity of amorphous phase

To estimate the volume fractions of the amorphous phase and side phases, the mass (M_{pellet}) and volume (V_{pellet}) of the pellets formed in the impedance cells were used. Based on the "actual" weight fractions of crystalline side phases m(side phase, i) obtained in RIR analysis and their theoretical densities ρ(side phase, i), the volume of each side phase i, V(side phase, i), can be obtained by following relationship:

$$V(\text{side phase}, i) = \frac{m(\text{side phase}, i) \cdot M_{\text{pellet}}}{\rho(\text{side phase}, i)}. \tag{3}$$

To enable the subsequent estimation, the amorphous phase and side phases were assumed to share the same relative density (D), taken as 90% for the calculation (i.e., a 10% volume fraction of pore in the pellet). The volume of the amorphous phase V(amorphous) was then obtained by

$$V(\text{amorphous}) = D \cdot V_{\text{pellet}} - \sum_{i=1}^{n} V(\text{side phase}, i). \tag{4}$$

The volume fraction of the amorphous phase, ϕ(amorphous), can be determined as

$$\phi(\text{amorphous}) = \frac{V(\text{amorphous})}{V_{\text{pellet}}}, \tag{5}$$

The volume fraction of the amorphous phase in $x_A Na_2O_{1.1}$-$TaCl_5$ (x_A = 0.82, 0.88, 1.03 and 1.55) was estimated and shown in Table S4.

Table S4 *The estimated volume fraction of amorphous phase in $x_A Na_2O_{1.1}$-$TaCl_5$ (x_A = 0.82, 0.88, 1.03 and 1.55), where NaCl was the only detected side phase. 10% volume fraction of pore was considered.*

	V_{pellet} / cm^3	M_{pellet} / g	ϕ(amorphous) / %
x_A = 0.82	0.0667	0.2005	79
x_A = 0.88	0.0667	0.2037	83
x_A = 1.03	0.0683	0.2003	75
x_A = 1.55	0.0824	0.1982	67

After obtaining the volume fractions, the effective conductivity can be estimated using the Bruggeman effective medium approximation,[15] by treating the side phase (NaCl) and pore as ionic insulators (σ = 0 S cm^{-1}). For the samples with x_A = 0.82 and 1.03, if the intrinsic ionic conductivity of the amorphous phase remained constant, the effective ionic conductivity would decrease by only ~9%. This value is far from sufficient to account for the experimentally observed nearly order-of-magnitude drop between those two samples, clearly indicating that the drop in ionic conductivity originates from a decrease in the intrinsic conductivity of the amorphous phase rather than from the change in the volume fraction of side phase(s).

9. Synchrotron X-ray total scattering patterns and $S(Q)$ data of $NaTaCl_6$ and $x_A Na_2O_{1.1}$-$TaCl_5$ (x_A = 0.52, 0.82, 1.03 and 1.55)

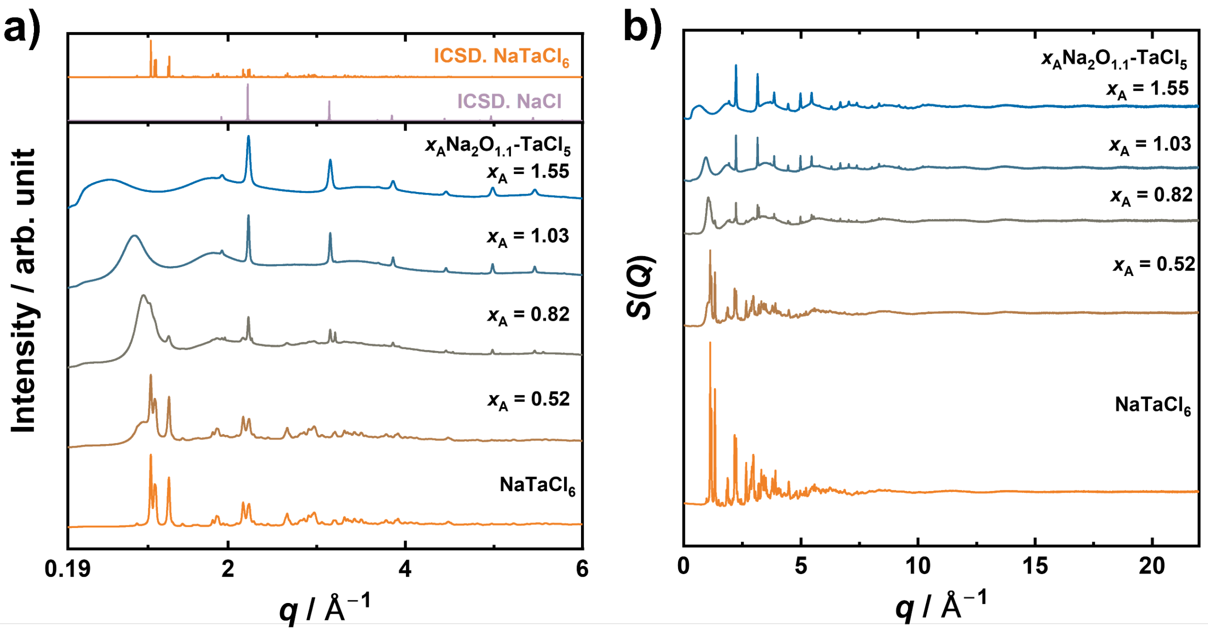

Figure S6 *(a) Synchrotron X-ray total scattering patterns of $x_A Na_2O_{1.1}$-$TaCl_5$ and (b) the corresponding structure factor S(Q). The pair distribution function G(r) in Figure 3 are converted from this S(Q).*

10. PDF analysis data of $x_A Na_2O_{1.1}$-$TaCl_5$ (x_A = 0.1, 0.21, 0.31) and NaCl

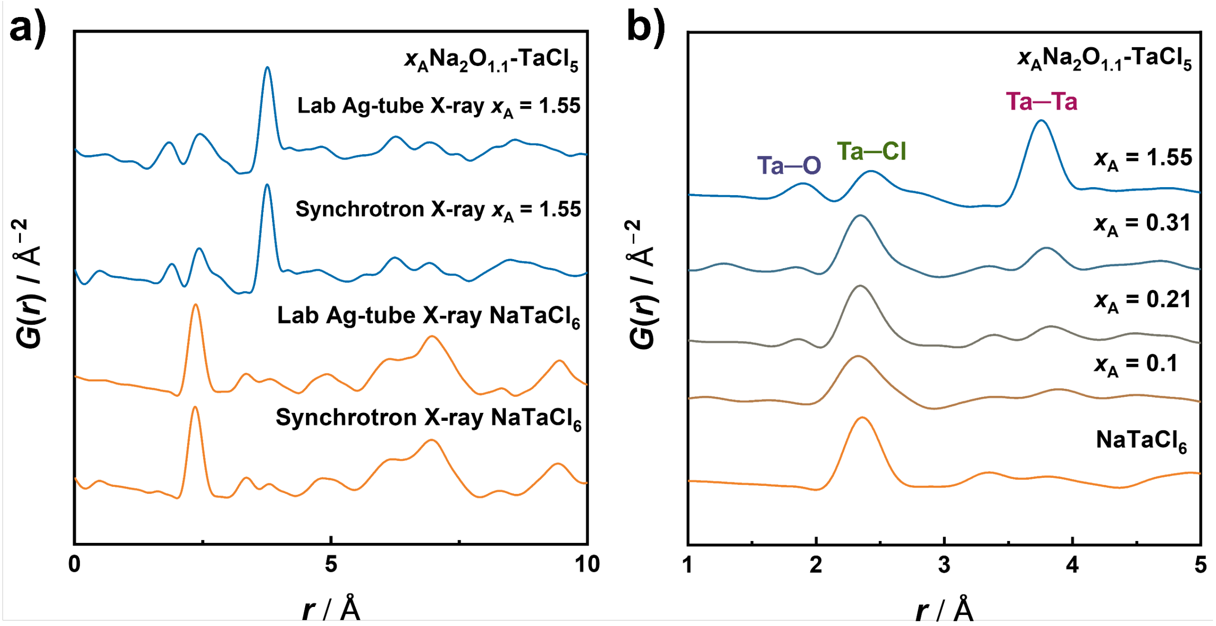

Figure S7 *(a) Comparison of the PDF analysis results for $NaTaCl_6$ and $1.55Na_2O_{1.1}$-$TaCl_5$ transformed from Synchrotron X-ray total scattering data and lab Ag-tube X-ray total scattering data. The PDF analysis results transformed from lab Ag-tube X-ray total scattering data capture the same structural features as those from Synchrotron X-ray total scattering data. (b) The PDF analysis results transformed from lab Ag-tube X-ray total scattering data, showing atomic distance changes that follow a similar trend to those observed in Figure 3.*

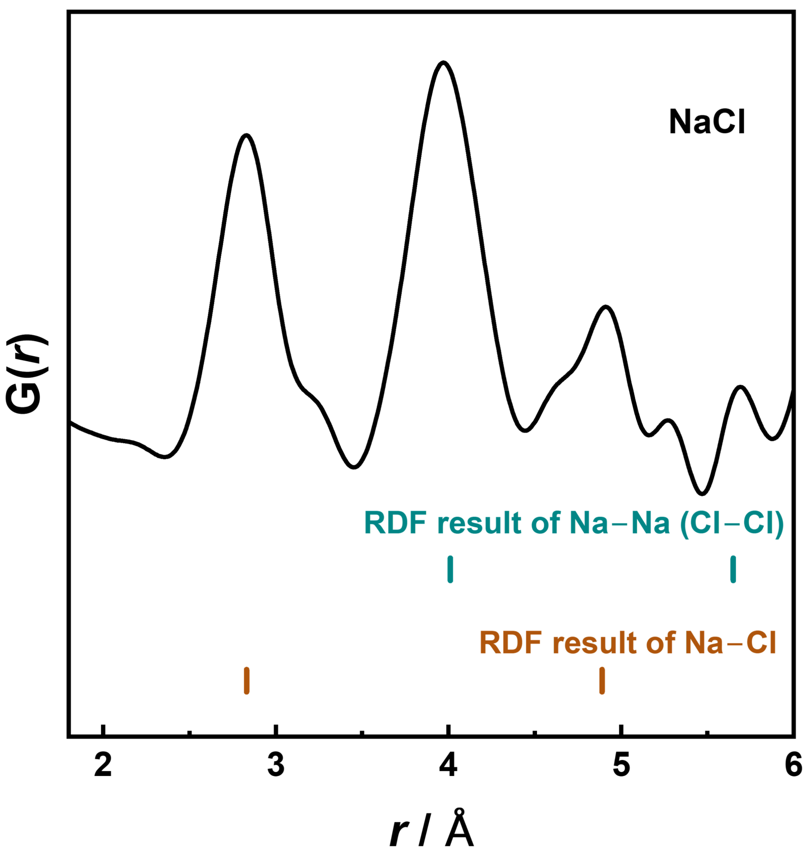

Figure S8 *Pair distribution function G(r) data of NaCl. The experiment observed intense peaks at 2.83 Å and 4.01 Å consistent with RDF results from CIF file of NaCl. Those peak positions are different from the discussed peaks in PDF patterns of* $x_A Na_2O_{1.1}$*-*$TaCl_5$ *and cannot be used to explain the peaks' shift in Figure 3 . Thus, the change in PDF data of* $x_A Na_2O_{1.1}$*-*$TaCl_5$ *is attributed to a local structure change in the amorphous phase.*

11. XRD, Raman spectroscopy, PDF analysis, and ion transport properties of samples synthesized using Na_2O_2 as precursor

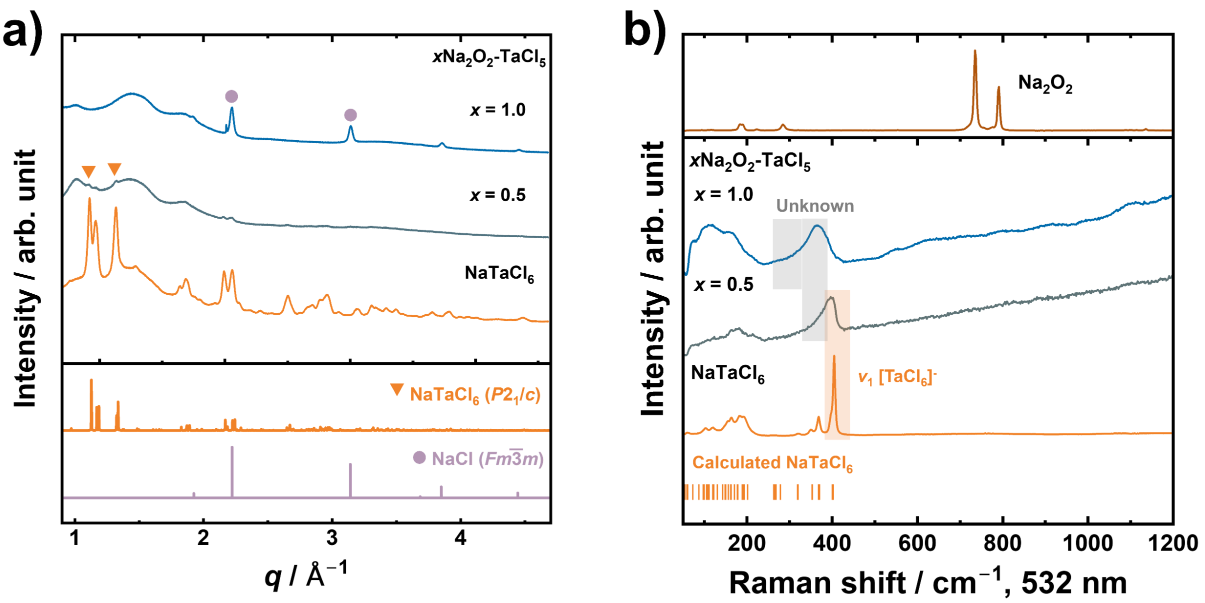

Figure S9 *(a) XRD patterns of xNa_2O_2-$TaCl_5$ (x = 0.5 and 1.0). For the sample with x = 0.5, weak peaks of $NaTaCl_6$ were detected. For sample incorporation with excess oxygen (x = 1.0), NaCl appeared as the side phase. These results suggest that the type of side phases is not influenced by whether Na_2O or Na_2O_2 is used as the precursor. (b) The Raman spectra of synthesized xNa_2O_2-$TaCl_5$, showing the same evolution as that observed in the $x_ANa_2O_{1.1}$-$TaCl_5$ series. No additional peaks were detected in Na_2O_2-derived samples, including those attributable to peroxide species in precursor Na_2O_2.*[16]

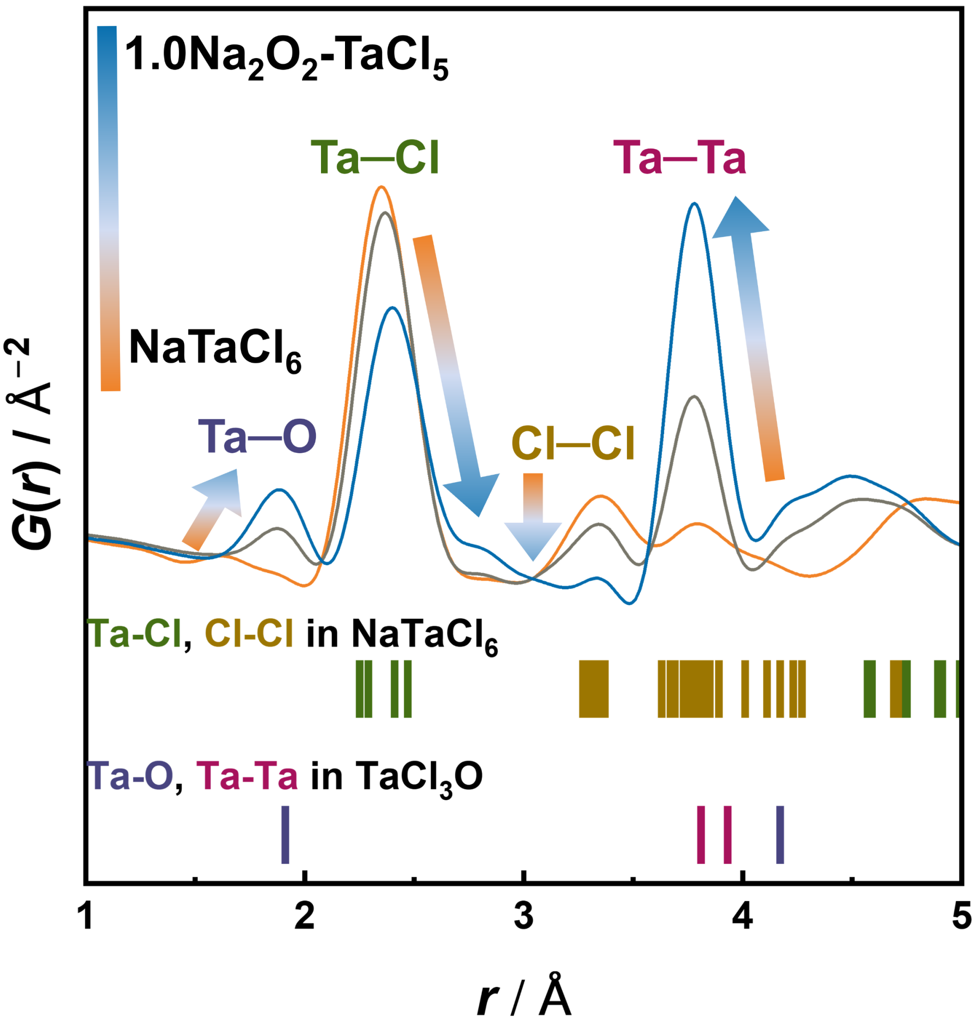

Figure S10 *PDF analysis results of synchrotron X-ray total scattering data of* $NaTaCl_6$ *and* xNa_2O_2-$TaCl_5$ *(x = 0.5 and 1.0), showing the same trend as that observed in* $x_ANa_2O_{1.1}$-$TaCl_5$ *series. No new peaks were observed compared with the* $x_ANa_2O_{1.1}$-$TaCl_5$ *series. Those results indicate that the choice of precursors (*Na_2O *or* Na_2O_2*) exerts little to no significant influence on the evolution of the local structure. PDF analysis results of* $0.5Na_2O_2$-$TaCl_5$ *more resemble those of* $0.52Na_2O_{1.1}$-$TaCl_5$ *rather than* $1.03Na_2O_{1.1}$-$TaCl_5$*, suggesting a likely release of oxygen from* Na_2O_2 *during ball milling.*

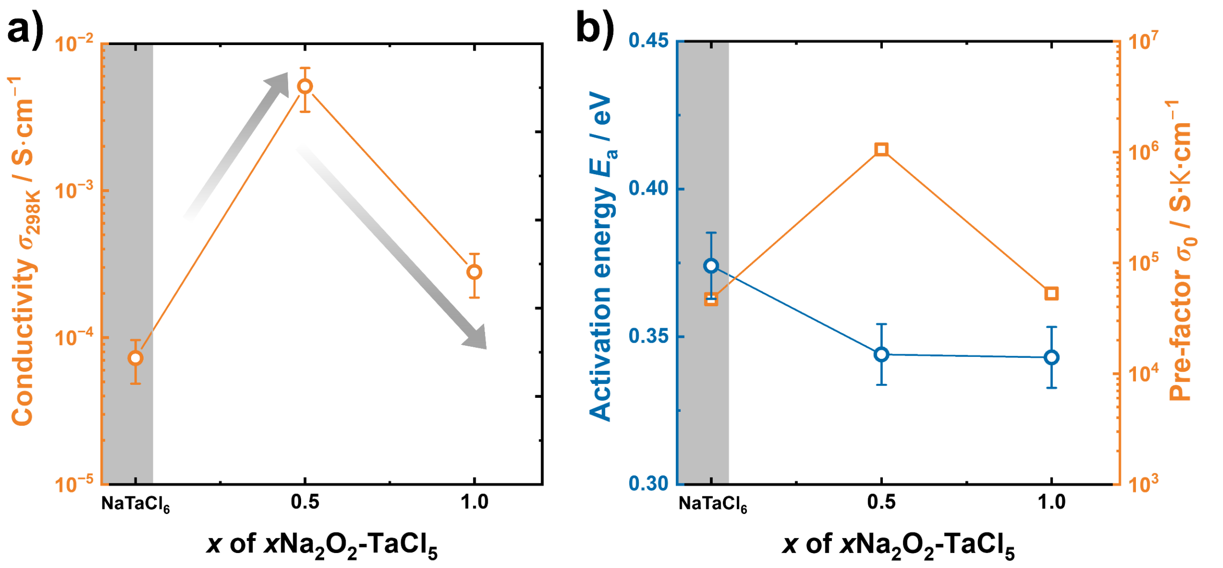

Figure S11 *(a) ionic conductivity and (b)activation energy and pre-factor. The error bars represent ±33 % of variation in ionic conductivity and ±3 % in activation energy. Trends are consistent with those in the* $x_A Na_2O_{1.1}$*-*$TaCl_5$ *series.*

To evaluate the potential influence of Na_2O_2 impurities in the Na_2O precursor, we synthesized the $x$$Na_2O_2$-$TaCl_5$ (x = 0.5 and 1.0) samples. The above experimental results indicate that the precursor (Na_2O or Na_2O_2) does not significantly affect the type of side phases, the evolution of local structures, and the overall trends observed in ion transport properties. However, the Raman spectrum and PDF analysis results of 0.5Na_2O_2-$TaCl_5$ more resemble those of 0.52$Na_2O_{1.1}$-$TaCl_5$ than those of 1.03$Na_2O_{1.1}$-$TaCl_5$, suggesting the possible oxygen release when using Na_2O_2 as the precursor. This also makes the one-to-one comparison between $x_A$$Na_2O_{1.1}$-$TaCl_5$ and $x$$Na_2O_2$-$TaCl_5$ more challenging. Nevertheless, considering that (i) Na_2O_2 accounts for only ~10 mol% in our Na_2O precursor, so even if oxygen release from Na_2O_2 occurs, its impact on the total oxygen content is minimal, and (ii) our focus is on the systematic changes induced by oxygen incorporation, which depend primarily on relative rather than absolute oxygen contents. Under these conditions, the discussed relationship between oxygen content, local structure, and ion transport properties in this work remains valid.

Table S5 *The activation energy, pre-factor, and ionic conductivity of synthesized samples*

Actual chemical formula	Ionic conductivity at 298 K / S cm^{-1}	Activation energy / eV	Pre-factor / S K cm^{-1}
0.5 Na_2O_2-$TaCl_5$	5.1(17) × 10^{-3}	0.344(17)	1.06 × 10^{6}
1.0 Na_2O_2-$TaCl_5$	2.8(9) × 10^{-4}	0.343(17)	5.27 × 10^{4}

12. XPS and XAS of samples

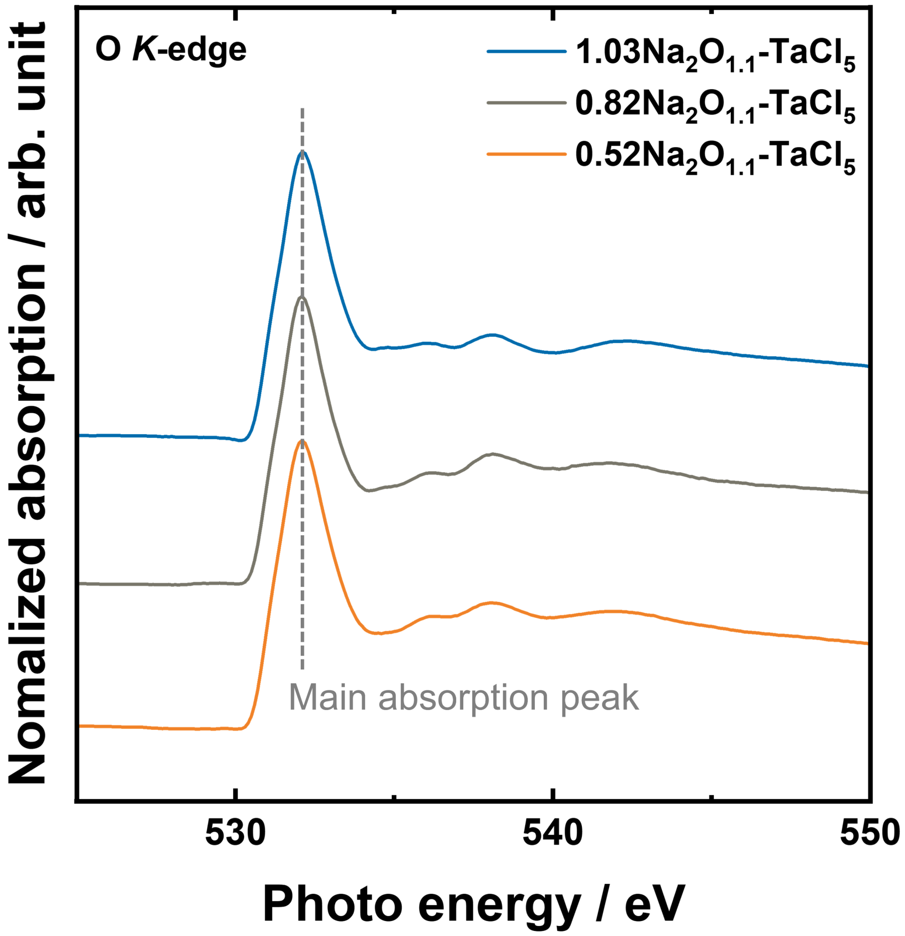

Figure S12 *O K-edge XAS spectra of 0.52*$Na_2O_{1.1}$*-*$TaCl_5$*, 0.82*$Na_2O_{1.1}$*-*$TaCl_5$*, and 1.03*$Na_2O_{1.1}$*-*$TaCl_5$*. Only one main absorption peak can be observed in XANES region, without apparent evolution in peak shape or position.*

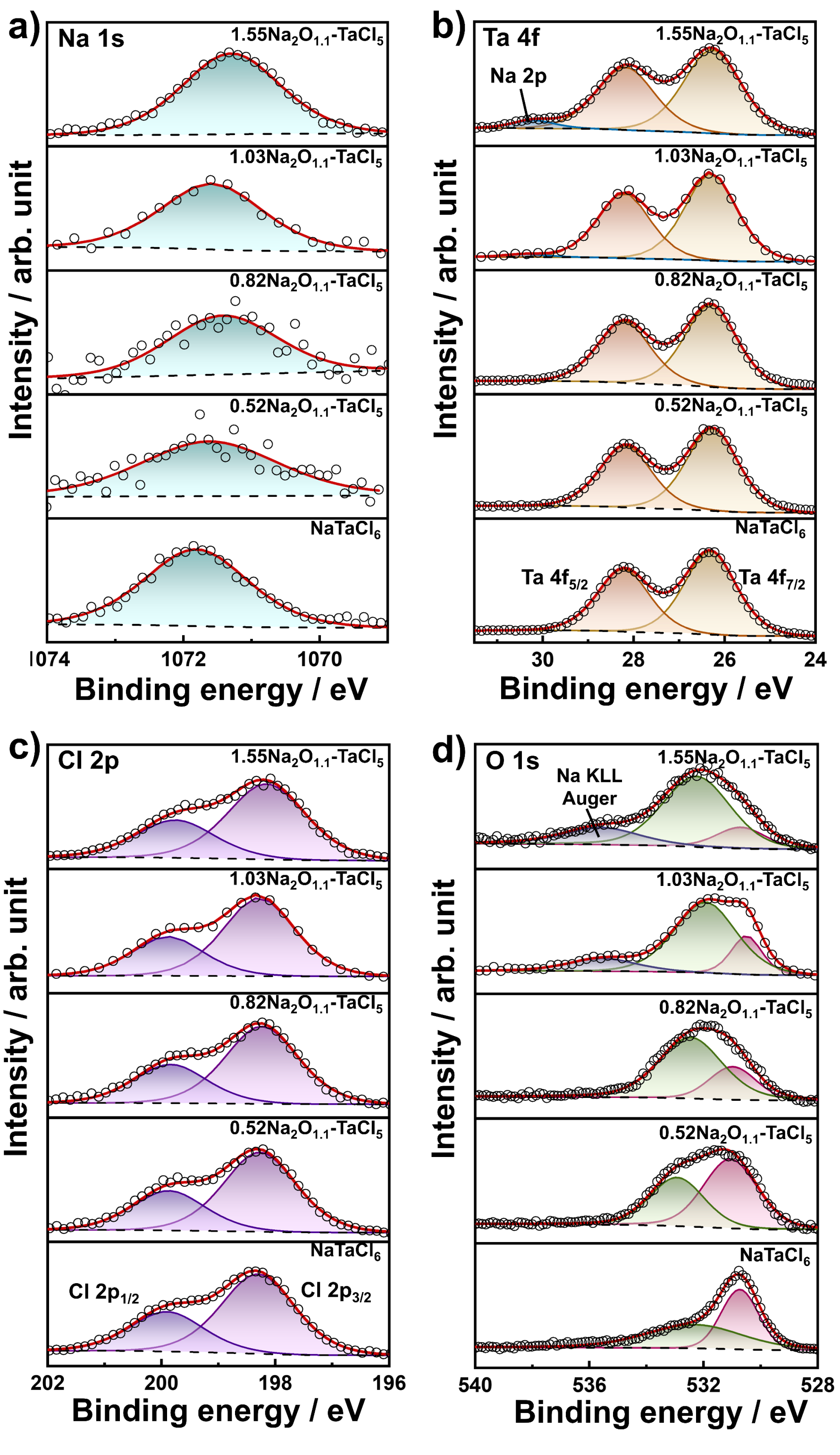

Figure S13 *XPS spectra of (a) Na 1s, (b) Ta 4f and (c) Cl 2p, and (d) O 1s for* $NaTaCl_6$ *and* $x_ANa_2O_{1.1}$-$TaCl_5$ *(*x_A *= 0.52, 0.82, 1.03, and 1.55). No apparent evolution in binding energy and full width at half maximum (FWHM) is observed for Na 1s, Ta 4f and Cl 2p. In contrast, the O 1s spectra vary markedly with oxygen content, indicating the coordination environment change of oxygen. The fitting of O 1s is shown only to highlight spectral changes, as the assignment remains challenging. The O 1s signal detected in* $NaTaCl_6$ *is likely attributable to adventitious oxygen contamination.*

13. Simulated local structure of xNa$_2$O-TaCl$_5$ (x = 0.5, 0.8, 1.0 and 1.5) and corresponding PDF analysis results

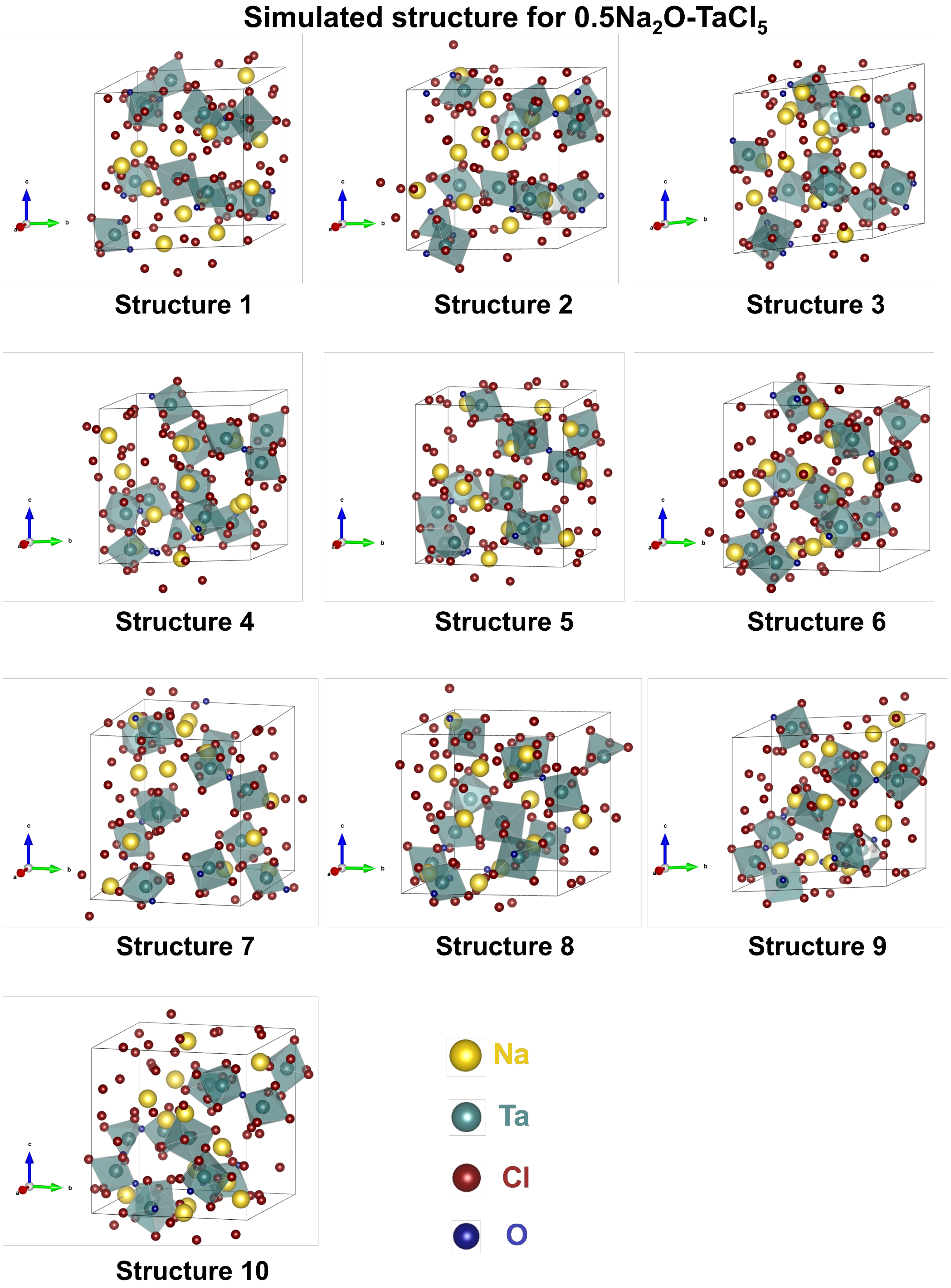

Figure S14 *Simulated structure for 0.5Na$_2$O-TaCl$_5$.*

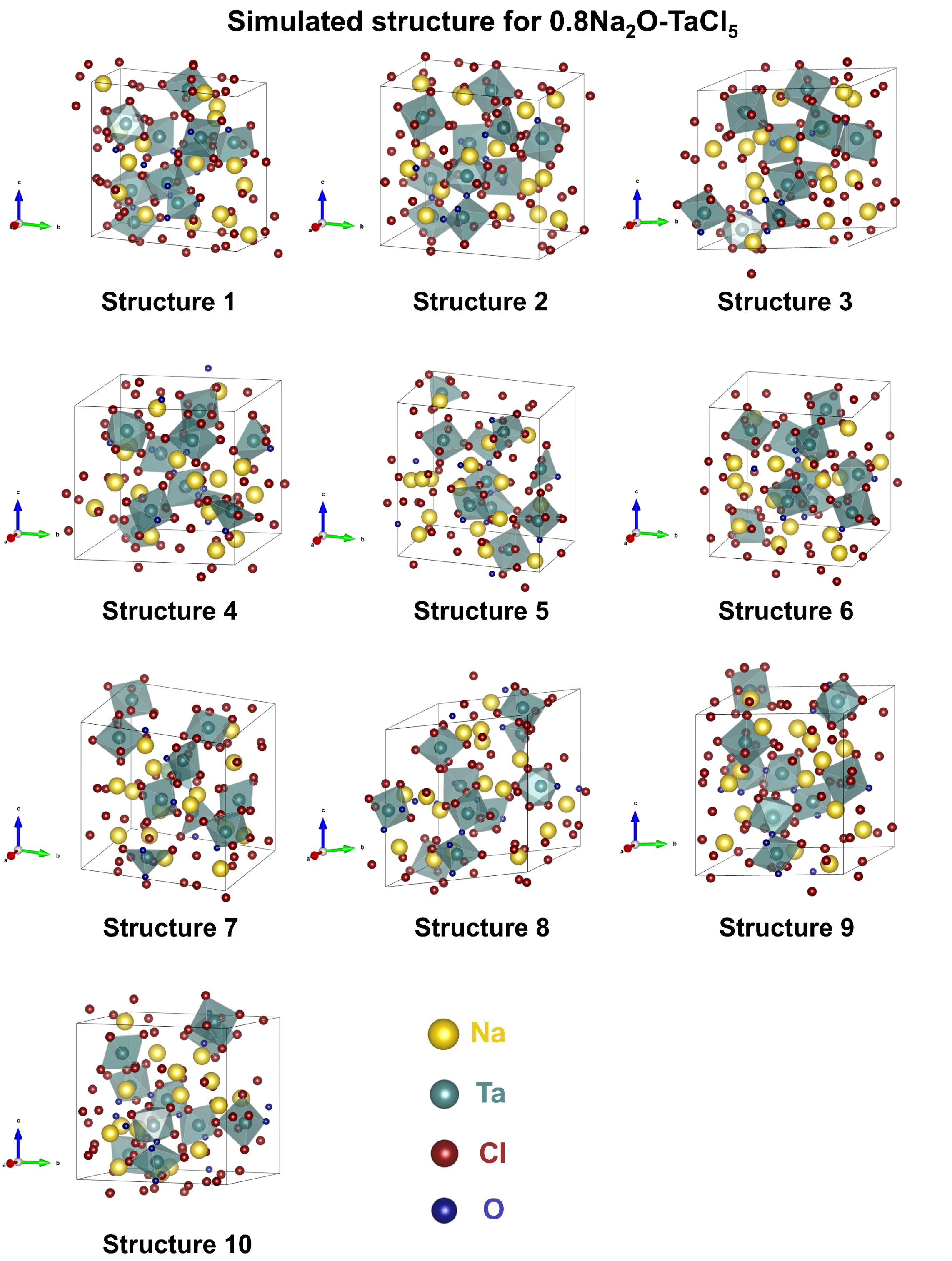

Figure S15 *Simulated structure for 0.8*Na_2O*-*$TaCl_5$*.*

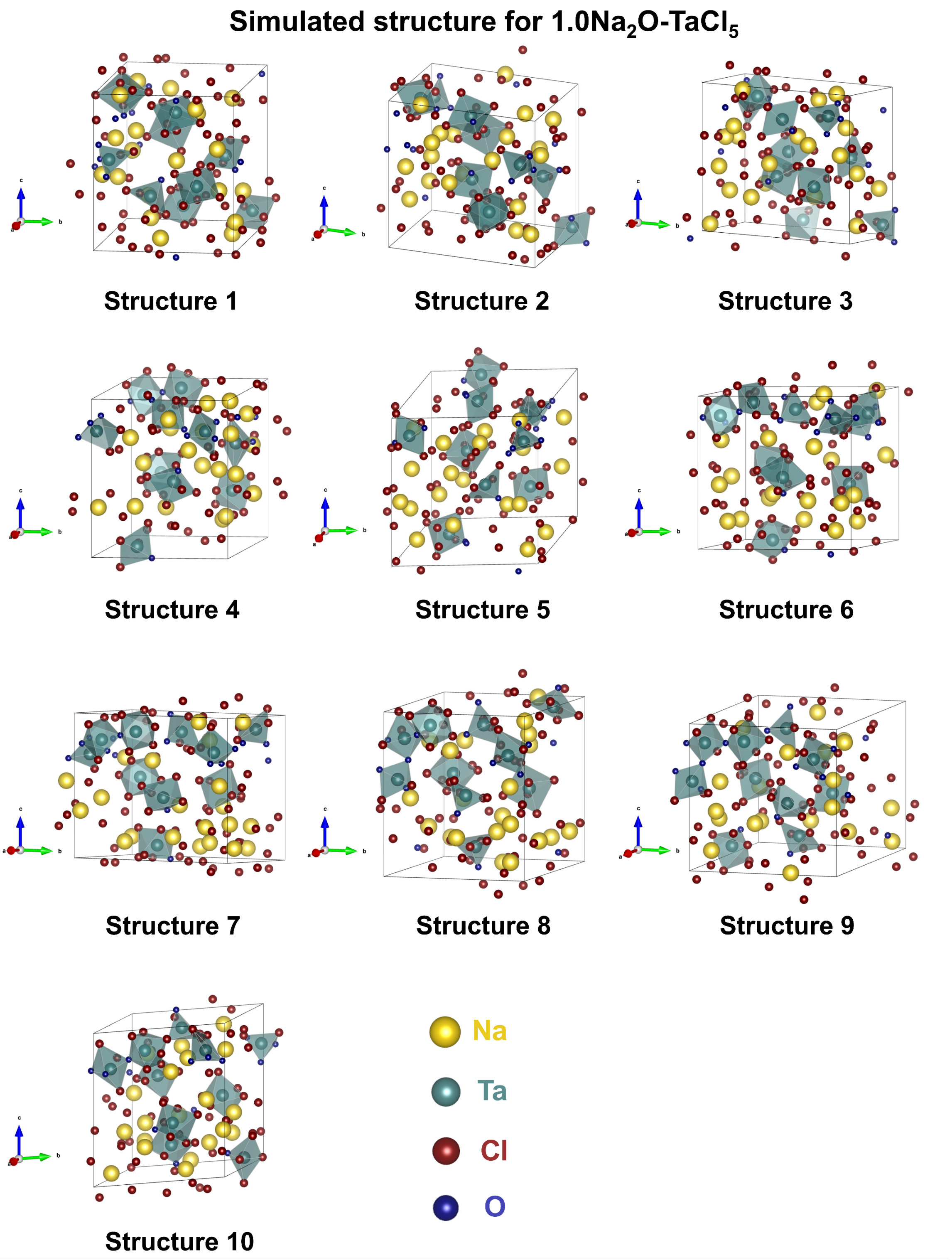

Figure S16 *Simulated structure for 1.0*Na_2O*-*$TaCl_5$*.*

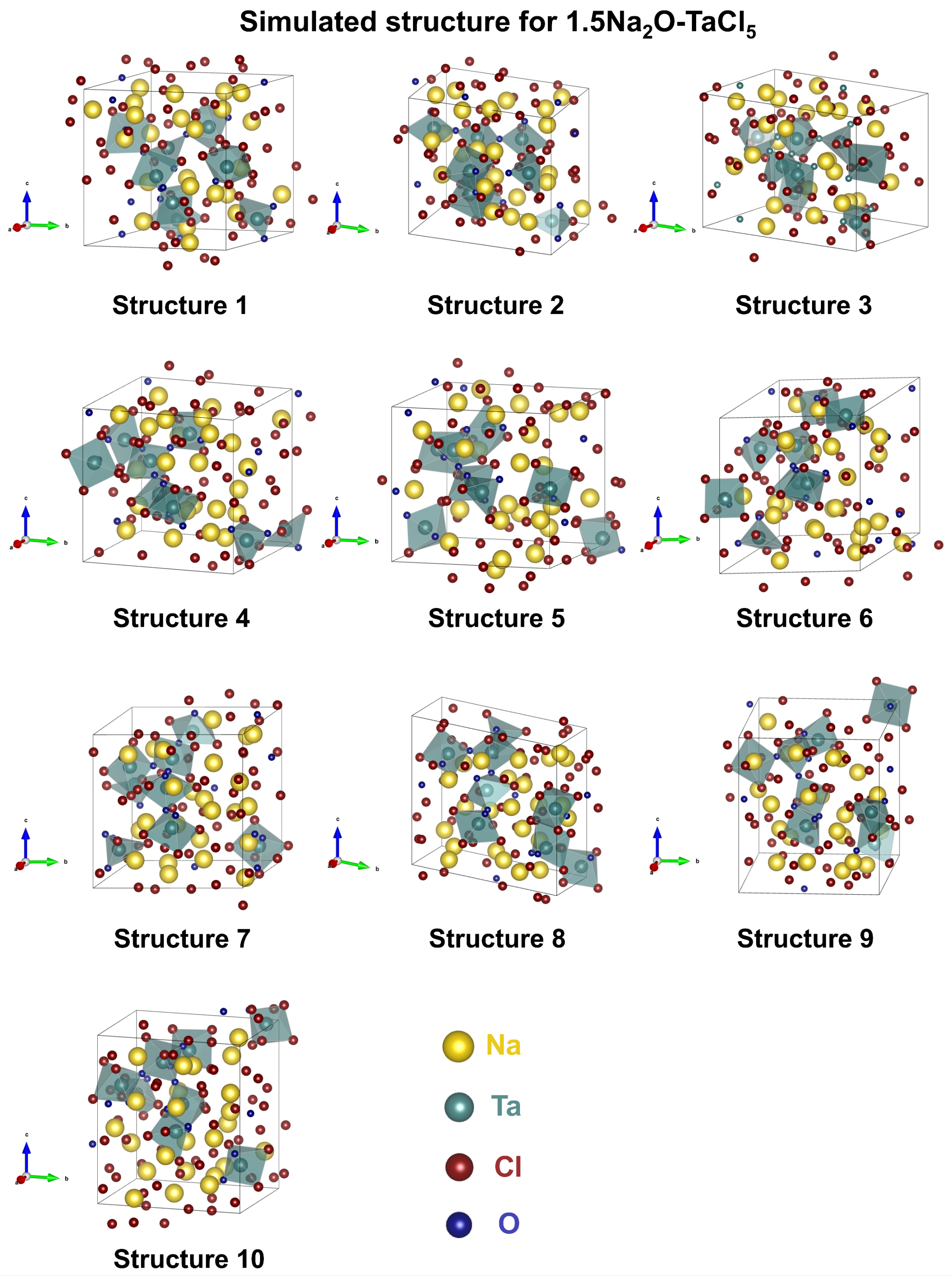

Figure S17 *Simulated structure for 1.5Na_2O-$TaCl_5$.*

__Table S6__ The ratio of bridging oxygen and non-bridging oxygen in modeled structures

$x_A Na_2O-TaCl_5$	Bridging oxygen / %	Bridging oxygen (average)	Corner oxygen / %	Corner oxygen (average)	Stoichiometry
$x_A = 0.5$	85	5.1	15	0.9	$Na_{12}Ta_{12}O_6Cl_{60}$
$x_A = 0.8$	44	3.5	56	4.5	$Na_{16}Ta_{10}O_8Cl_{50}$
$x_A = 1.0$	35	3.5	65	6.5	$Na_{20}Ta_{10}O_{10}Cl_{50}$
$x_A = 1.5$	35	4.2	65	7.8	$Na_{24}Ta_8O_{12}Cl_{40}$

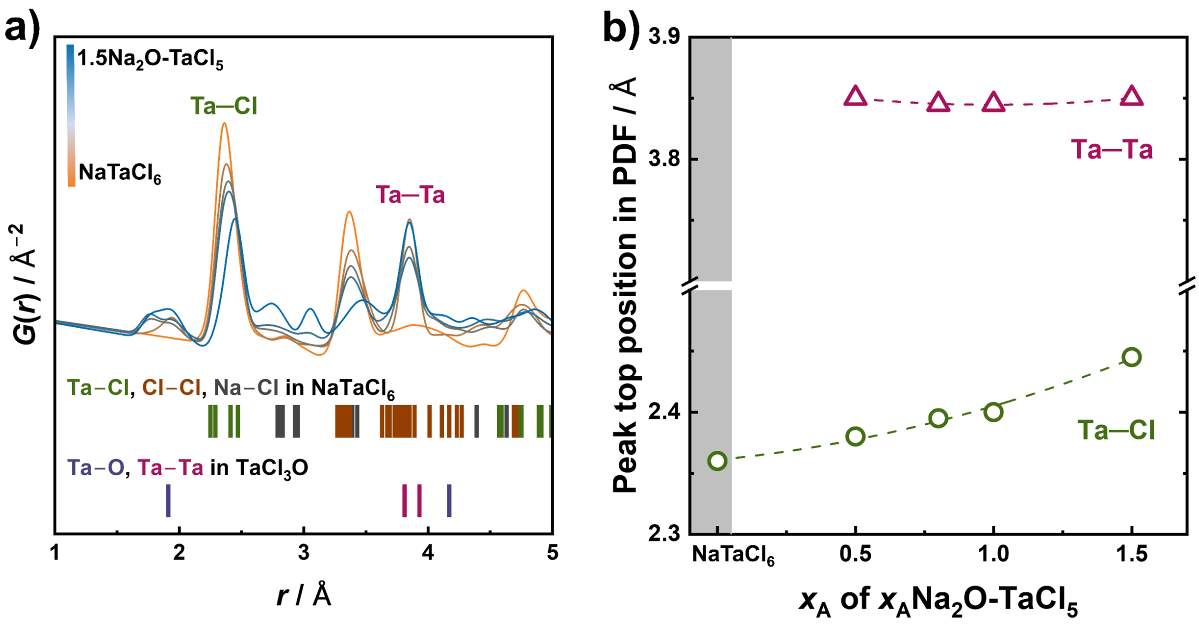

***Figure S18** (a)PDF data transferred from simulated structures and (b)the corresponding peak top position in PDF plot.*

References

(1) Kızılaslan, A.; Çelik, M.; Fujii, Y.; Huang, Z.; Moriyoshi, C.; Kawaguchi, S.; Hiroi, S.; Ohara, K.; Ando, M.; Tadanaga, K.; Ohno, S.; Miura, A. The Detail Matters: Unveiling Overlooked Parameters in the Mechanochemical Synthesis of Solid Electrolytes. *ACS Energy Lett*. **2025**, 10(1), 156–160.

(2) Momma, K.; Izumi, F. VESTA 3 for Three-Dimensional Visualization of Crystal, Volumetric and Morphology Data. *J. Appl. Crystallogr.* **2011**, *44* (6), 1272–1276.

(3) Cohelo, A. *TOPAS-Academic: Coelho Software*; Brisbane, **2007**.

(4) Ohara, K.; Onodera, Y.; Murakami, M.; Kohara, S. Structure of Disordered Materials under Ambient to Extreme Conditions Revealed by Synchrotron X-Ray Diffraction Techniques at SPring-8 - Recent Instrumentation and Synergic Collaboration with Modelling and Topological Analyses. *J. Condens. Matter*. *Phys.* **2021**, *33*, 383001.

(5) Lorch, E. Neutron Diffraction by Germania, Silica and Radiation-Damaged Silica Glasses. *J. Phys. C: Solid State Phys.* **1969**, *2*, 229.

(6) Martinez, L.; Andrade, R.; Birgin, E. G.; Martínez, J. M. PACKMOL: A Package for Building Initial Configurations for Molecular Dynamics Simulations. *J. Comput. Chem.* **2009**, *30* (13), 2157–2164.

(7) Kresse, G.; Furthmiiller, J. Efficiency of Ab-Initio Total Energy Calculations for Metals and Semiconductors Using a Plane-Wave Basis Set. *Comput. Mater. Sci.* **1996**, *6*, 15.

(8) Kresse, G.; Furthmü, J. Efficient Iterative Schemes for Ab Initio Total-Energy Calculations Using a Plane-Wave Basis Set. *Phys. Rev. B* **1996**, *54*, 11169.

(9) Blöchl, P. E. Projector Augmented-Wave Method. *Phys. Rev. B* **1994**, *50* (24), 17953–17979.

(10) Perdew, J. P.; Burke, K.; Ernzerhof, M. Generalized Gradient Approximation Made Simple. *Phys. Rev. Lett.* **1996**, *77*, 3865.

(11) d'Avezac, M.; Graf, P.; Paudal, T.; Peng, H.; Zhang, L.; Stephen, S.; Stevanovic, V. Pylada: a comprehensive python framework for preparing, running, monitoring, analyzing, and archiving high throughput first principles calculations. *GitHub repository* **2010**.

(12) Stukowski, A. Visualization and Analysis of Atomistic Simulation Data with OVITO-the Open Visualization Tool. *Model. Simul. Mater. Sci. Eng.* **2010**, *18* (1).

(13) Warren, B. E.; Krutter, H.; Morningstar, O. Fourier Analysis of X-ray Patterns of Vitreous SiO_2 and B_2O_2*. *J. Am. Ceram. Soc.* **1936**, *19* (1–12), 202–206.

(14) Lin, X.; Zhang, S.; Yang, M.; Xiao, B.; Zhao, Y.; Luo, J.; Fu, J.; Wang, C.; Li, X.; Li, W.; Yang, F.; Duan, H.; Liang, J.; Fu, B.; Abdolvand, H.; Guo, J.; King, G.; Sun, X. A Family of Dual-Anion-Based Sodium Superionic Conductors for All-Solid-State Sodium-Ion Batteries. *Nat. Mater.* **2025**, *24* (1), 83–91.

(15) Bruggeman, D. A. G. Berechnung Verschiedener Physikalischer Konstanten von Heterogenen Substanzen. I. Dielektrizitätskonstanten Und Leitfähigkeiten Der Mischkörper Aus Isotropen Substanzen. *Ann. Phys.* **1935**, *416* (7), 636–664.

(16) Ohtori, N.; Ueno, F.; Furukawa, T. Raman Spectra of Peroxide Ions at Hight Temperature.

Electrochem. **2005**, *73* (8), 597–599.
